# Current Concepts on the Reno-Protective Effects of Phosphodiesterase 5 Inhibitors in Acute Kidney Injury: Systematic Search and Review

**DOI:** 10.3390/jcm9051284

**Published:** 2020-04-29

**Authors:** Georgios Georgiadis, Ioannis-Erineos Zisis, Anca Oana Docea, Konstantinos Tsarouhas, Irene Fragkiadoulaki, Charalampos Mavridis, Markos Karavitakis, Stavros Stratakis, Kostas Stylianou, Christina Tsitsimpikou, Daniela Calina, Nikolaos Sofikitis, Aristidis Tsatsakis, Charalampos Mamoulakis

**Affiliations:** 1 Department of Urology, University General Hospital of Heraklion, University of Crete, Medical School, Heraklion, Crete, Greece; geokosgeo@yahoo.gr (G.G.); renoszisis@gmail.com (I.-E.Z.); eirinimbg@hotmail.gr (I.F.); ch.mavridis@uoc.gr (C.M.); markoskaravitakis@yahoo.gr (M.K.); 2 Department of Forensic Sciences and Toxicology, Faculty of Medicine, University of Crete, Heraklion, Crete 71003, Greece; tsatsaka@uoc.gr; 3 Department of Toxicology, University of Medicine and Pharmacy of Craiova, 200349 Craiova, Romania; daoana00@gmail.com; 4 Department of Cardiology, University General Hospital of Larissa, Larissa, Greece; ktsarouhas14@yahoo.gr; 5 Department of Nephrology, University General Hospital of Heraklion, University of Crete, Medical School, Heraklion, Crete, Greece; stratakisst@gmail.com (S.S.); kstylianu@gmail.com (K.S.); 6 Department of Hazardous Substances, Mixtures and Articles, General Chemical State Laboratory of Greece, Ampelokipi, Athens, Greece; chtsitsi@yahoo.com; 7 Department of Clinical Pharmacy, University of Medicine and Pharmacy of Craiova, 200349 Craiova, Romania; calinadaniela@gmail.com; 8 Department of Urology, School of Medicine, Ioannina University, Ioannina, Greece; akrosnin@hotmail.com

**Keywords:** acute kidney injury, avanafil, icariin, phosphodiesterase 5 inhibitors, renal insufficiency, sildenafil citrate, tadalafil, udenafil, vardenafil dihydrochloride, zaprinast

## Abstract

Acute kidney injury (AKI) is associated with increased morbidity, prolonged hospitalization, and mortality, especially in high risk patients. Phosphodiesterase 5 inhibitors (PDE5Is), currently available as first-line therapy of erectile dysfunction in humans, have shown a beneficial potential of reno-protection through various reno-protective mechanisms. The aim of this work is to provide a comprehensive overview of the available literature on the reno-protective properties of PDE5Is in the various forms of AKI. Medline was systematically searched from 1946 to November 2019 to detect all relevant animal and human studies in accordance with the Preferred Reporting Items for Systematic Reviews and Meta-analyses (PRISMA) statement. In total, 83 studies were included for qualitative synthesis. Sildenafil is the most widely investigated compound (42 studies), followed by tadalafil (20 studies), icariin (10 studies), vardenafil (7 studies), zaprinast (4 studies), and udenafil (2 studies). Even though data are limited, especially in humans with inconclusive or negative results of only two clinically relevant studies available at present, the results of animal studies are promising. The reno-protective action of PDE5Is was evident in the vast majority of studies, independently of the AKI type and the agent applied. PDE5Is appear to improve the renal functional/histopathological alternations of AKI through various mechanisms, mainly by affecting regional hemodynamics, cell expression, and mitochondrial response to oxidative stress and inflammation.

## 1. Introduction

AKI is considered a complex disorder with increased morbidity, prolonged hospitalization and mortality especially in high risk patients that may be attributed to various causes (pre-renal; renal, i.e., intrinsic to the renal parenchyma; and post-renal), including the use of nephrotoxic medications such as contrast media (CM), dehydration, sepsis, renal surgery, renal ischemia, ischemia–reperfusion (IR) renal injury, and urinary tract obstruction [1]. Criteria used for the diagnosis of AKI vary widely among studies in humans [2], including percent change in the baseline serum creatinine (sCr) levels (e.g., an increase of variously 25–50%) and absolute elevation from baseline sCr level (e.g., an increase of variously 0.5–2.0 mg/dL) [3]. These variable definitions have been addressed by two consensus groups, namely the Acute Dialysis Quality Initiative (ADQI) proposing the RIFLE (Risk, Injury, Failure, Loss and End-stage kidney disease) system [4] and more recently the Acute Kidney Injury Network (AKIN), which have attempted to standardize the diagnosis of AKI irrespective of etiology. According to the AKIN diagnostic criteria [5], AKI is an abrupt (within 48 h) reduction in human kidney function defined as occurrence of any of the following after a reno-toxic event: (a) absolute increase in sCr ≥ 0.3 mg/dL (≥ 26.4 μmol/L) or a percentage increase in sCr ≥ 50% (1.5-fold from baseline), which is known or presumed to have occurred within the prior seven days [6]; or (b) a reduction in urine output (documented oliguria of < 0.5 mL/kg/h for more than 6 h). This definition is in accordance with the current Clinical Practice Guideline for AKI by “Kidney Disease: Improving Global Outcomes” (KDIGO) [6]. Nevertheless, a recent systematic review evaluating the methods used to investigate AKI biomarkers showed that results are difficult to interpret, not comparable, and not consistently reproducible due to the impact of the variable AKI definitions still used to determine the outcome of interest in human studies (38.0% of the studies used the AKIN; 21.4% used the RIFLE; 20.3% used the KDIGO; and 20.3% used another definition) [2]. Similarly, variable definitions of AKI have been used in animal studies, a fact that has been recognized as an important limitation in translating preclinical findings in clinical studies [7,8] among others [9]. Several reviews of available animal models, including their advantages and disadvantages, have been discussed [10]; however, the types of models are often incomplete and many details, such as model techniques and modeling time, are not mentioned. Currently proposed AKI models include, among others: IR renal injury, including shock wave lithotripsy (SWL); injection of drugs, toxins, or endogenous toxins; ureteral obstruction, contrast-induced nephropathy (CIN); trauma such as burn; etc. [10,11,12,13,14,15,16].

Depending on the insult type, there are various mechanisms leading to renal damage such as renal vasoconstriction [17], vascular endothelial damage, cytokine expression [18], increase of IL-18, mediating acute tubular necrosis, caspase activity stimulation, p53 up-regulation [19], accumulation of toxic metabolites [20], mast cells/neutrophils activation, reactive oxygen species (ROS) generation causing lipid peroxidation that leads to cellular membrane destruction, excessive intracellular DNA breakdown, energy depletion, intracellular Ca2+ elevation, higher inducible nitric oxide (NO) synthase (iNOS) expression, NO deficiency, intra-parenchymal hemorrhage [21], fibrosis, direct cellular toxicity, tubular obstruction, vascular congestion, activation of angiotensin II axis [22], mitochondrial dysfunction [23], cell cycle arrest in G2 phase, ATPase activity inhibition, and cellular transport modification. ROS activate pro-apoptotic proteins eventually promoting Bax translocation (regulated by PI3K/Akt pathway) to the outer mitochondrial membrane, causing the release of cytochrome c in the cytosol [24]. Bax is also responsible for caspase 9 activation that activates caspase 3, triggering apoptosis. The tubular component of AKI consists of injured, necrotic/apoptotic cells falling into the lumen that cause obstruction/back leak of the filtrate to the interstitial space, inducing inflammation.

CIN is a real, albeit rare, entity in current clinical medical practice that represents a serious iatrogenic AKI form, occurring 24–72 h after administration of iodinated contrast media (CM) during angiographic or other procedures, such as urography [3,25]. The exact pathophysiology of CIN is not fully elucidated but oxidative stress is considered a major mechanism in CIN [26], and the identification of novel biomarkers that may more accurately detect renal function changes, reflect kidney damage, assist monitoring, and elucidate pathophysiology have attracted considerable scientific attention nowadays [27]. CM passing through the kidney results in an intense tubular transport that increases the activity in the thick ascending limb of Henle’s loop. This process increases oxygen consumption/metabolic activity of outer renal medulla, exacerbating the marginal hypoxic conditions. Prostanoids and NO are mainly responsible for the medullary vasodilatory response [28]. Therefore, any NO deficit may contribute to an additional hypoxic renal insult. CIN and IR renal injury share common pathways regarding the vasodilatory potential of NO. IR renal injury is a common complication during renal transplantation/artery angioplasty, partial nephrectomy, cardiopulmonary/aortic bypass surgery, and others [29]. In the IR renal injury setting, however, there are conflicting results reported, with some studies suggesting that NO induces cytotoxicity, and others showing that increased NOS activity is linked to increased renal blood flow in the ischemic region [30]. NOSs are a family of enzymes catalyzing the production of NO from L-arginine. There are three isoforms: the endothelial NOS (eNOS), the neuronal NOS (nNOS), and the iNOS involved in immune response. In the IR renal injury, endogenous NO is synthesized by eNOS and iNOS [31], while it is found that eNOS-mediated NO production plays a pivotal protective role in IR-induced AKI [1]. IR renal injury is also closely linked to ROS generation/apoptosis.

**Figure 1:**
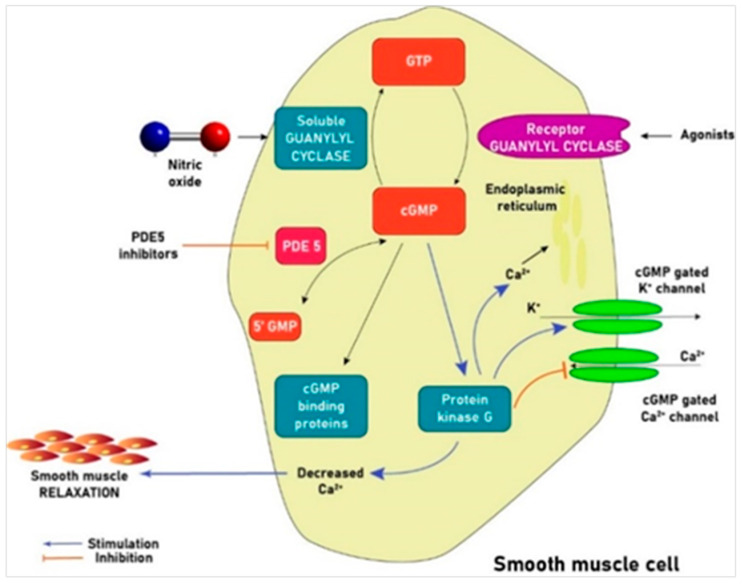
PDE5I-induced smooth muscle relaxation in the corpora cavernosa. cGMP is the principal mediator of NO-induced smooth muscle relaxation/vasodilation [35]. cGMP propels a series of intracellular changes including inhibition of Ca^2+^ entry into the cell, Ca^2+^ shift into the endoplasmic reticulum, activation of K^+^ channels leading to membrane hyperpolarization, and stimulation of a cGMP-dependent protein kinase that activates a myosin light chain phosphatase. All these actions promote smooth muscle relaxation. NO penetrates the cytoplasm of smooth muscle cells binding to guanylyl cyclase (sGC), which catalyzes the enzymatic conversion of GTP to cGMP. Elevation of cGMP stimulates cGMP-dependent protein kinase G leading to PDE5 phosphorylation/activation. PDE5 hydrolyzes cGMP in the cavernosal tissue. Inhibition of PDE5 results in smooth muscle relaxation with increased arterial blood flow, leading to compression of the sub-tunical venous plexus followed by penile erection [36].

Prevention and/or management of the various AKI forms, such as CIN, is mainly supportive at present, consisting of intravenous hydration [32]. Even though the potential beneficial effects of many agents with antioxidant properties have been tested, the value of such substances other than sodium bicarbonate remains controversial [32,33]. Phosphodiesterase 5 (PDE5) inhibitors (PDE5Is) are currently recommended as first-line therapy of erectile dysfunction (ED) by enhancing the vasodilatory effects of NO [34]. Acting via the selective inhibition of cyclic guanosine monophosphate (cGMP)-specific PDE5 that metabolizes cGMP, the principal mediator of NO-induced smooth muscle relaxation, PDE5Is cause vasodilatation in the corpora cavernosa promoting erection (Figure 1). This class of drugs has shown beneficial potential through various mechanisms in some CIN animal models [33]. The aim of this paper is to provide a comprehensive overview of the available literature on the potential reno-protective properties of PDE5Is in the various forms of AKI.

## 2. Experimental Section

Medline (Ovid Medline Epub Ahead of Print, In-Process & Other Non-Indexed Citations, Ovid MEDLINE(R) Daily, and Ovid MEDLINE(R) 1946 to November 2019) was systematically searched to detect all relevant animal and human studies in accordance with the Preferred Reporting Items for Systematic Reviews and Meta-analyses (PRISMA) statement [37], using the following keyword combinations (Medical Subject Headings; MeSH): PDE5i or avanafil or benzamidenafil or dasantafil or icariin or lodenafil or mirodenafil or sildenafil or tadalafil or udenafil or vardenafil or zaprinast combined with renal or kidney or nephrotoxicity or contrast or CIN or AKI or nephrotoxic or cisplatin or aminoglycoside or trauma or acute kidney injury or NSAIDS or non-steroidal or shock or sepsis or hypoperfusion or hypovolaemia or hypovolemia or renal artery stenosis or obstruction or acute tubular necrosis or glomerulonephritis or nephritis or renal failure or adenine or cyclosporine. The specific literature search strategy used is available in Appendix A. The reference lists of selected studies were screened for other potentially eligible studies. After excluding duplicates, citations in abstract form, and non-English citations, the titles/abstracts of full papers were screened for relevance, defined as original research focusing on the topic “nephropathy AND effects of phosphodiesterase 5 inhibitors”. Studies focusing on alterations of renal function and/or structure for >3 months (conventionally considered as following the KDIGO definition of chronic kidney disease (CKD) were excluded [6]). Two review authors (G.G. and IE.Z.) independently scanned the title and the abstract content, or both, of every record retrieved to determine which studies should be assessed further evaluated and extracted all data. Disagreements were resolved through consensus or by consultation with a third author (C.M.). A final draft of the manuscript was prepared after several revisions and approved by all authors.

## 3. Results

**Figure 2:**
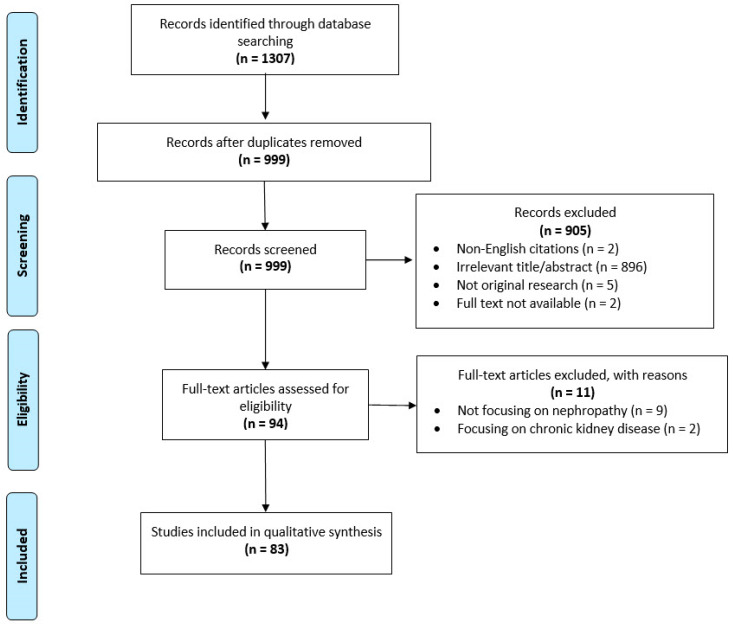
PRISMA flow chart showing the study selection procedure.

In total, 83 studies were included for qualitative synthesis (Figure 2). Among the 11 natural/synthetic agents currently available (avanafil, benzamidenafil, dasantafil, icariin, lodenafil, mirodenafil, sildenafil, tadalafil, udenafil, vardenafil, and zaprinast), sildenafil is the most widely investigated (n = 42 studies), followed by tadalafil (n = 20 studies), icariin (n = 10 studies), vardenafil (n = 7 studies), zaprinast (n = 4 studies), and udenafil (n = 2 studies). No studies on lodenafil, benzamidenafil, mirodenafil, avanafil, or dasantafil were detected. Most of the studies (n = 79) used animal models, including among others currently proposed AKI models (IR renal injury, including SWL; injection of drugs, toxins, or endogenous toxins; ureteral obstruction; CIN; trauma such as burn; etc.) [10,11,12,13,14,15,16] and variable definitions of AKI in line with the situation observed in human studies [2]. Only four human studies were detected: two preclinical studies utilizing human tissue [24,38] and two clinical trials [17,39].

The reno-protective action of PDE5Is was evident in the vast majority of studies (n = 81), independently of the AKI type and the agent applied. Only one human study on sildenafil [39] and one animal study on zaprinast [40] failed to reveal any reno-protective action of PDE5Is, showing a neutral effect. PDE5Is appeared to be beneficial in AKI of various etiologies by improving renal functional/histopathological alternations through various mechanisms, such as affecting regional hemodynamics, cell expression, and mitochondrial response to oxidative stress and inflammation.

The main characteristics and results of the human studies evaluating the potential reno-protective effects of PDE5Is are summarized in Table 1 [17,24,38,39]. The main characteristics and results of the animal studies on currently proposed AKI models evaluating the potential reno-protective effects of sildenafil, tadalafil, icariin, vardenafil, zaprinast–udenafil are summarized in Table 2 [23,30,41,42,43,44,45,46,47,48,49,50,51,52,53,54,55,56,57,58,59,60,61], Table 3 [29,35,45,49,62,63,64,65,66,67,68,69,70,71,72,73,74], Table 4 [18,75,76], Table 5 [45,77,78], and Table 6 [21,40,79,80], respectively. The main characteristics and results of the animal studies in the AKI-CKD transition spectrum (focusing on renal function and/or structure alterations for up to three months, not fulfilling the KDIGO definition for CKD [6]) evaluating the potential reno-protective effects of sildenafil, tadalafil, icariin, vardenafil, zaprinast–udenafil are summarized in Table A1 [19,81,82,83,84,85,86,87,88,89,90,91,92,93,94,95,96,97,98], Table A2 [99,100], Table A3 [22,101,102,103,104], Table A4 [105,106,107,108], and Table A5 [109,110], respectively (Appendix B).

## 4. Discussion

PDE5Is have received a lot of attention since the first drugs were launched in the market. Four potent selective agents (avanafil, sildenafil, tadalafil, and vardenafil) have been approved by the European Medicines Agency (EMA) and the Food and Drug Administration (FDA) for the treatment of ED [111,112]. ED can be managed successfully with currently available treatment options, but it cannot be cured and most patients will be treated without cause-specific options, such as the use of PDE5Is [34]. Exceptions are psychogenic, post-traumatic arteriogenic in young patients, and hormonal causes (e.g., hypogonadism) of ED, which are potentially curable with specific treatments that might be employed first, when such causes are detected [34]. Consequently, treatment strategy of ED should be tailored depending on invasiveness, efficacy, safety, cost, and patient preference of the currently available options; in the context of this strategy, PDE5Is are currently recommended strongly as first-line treatment option given that lifestyle changes are initiated/risk factors are modified prior to or at the same time as initiating ED treatment [34].

Other EMA/FDA approved indications of PDE5Is include pulmonary arterial hypertension (PAH) (sildenafil and tadalafil) and management of men with moderate to severe LUTS secondary to benign prostatic obstruction with or without ED (tadalafil) [34,113,114,115]. Besides the aforementioned agents, there are other non-EMA/FDA approved PDE5Is including benzamidenafil, dasantafil, lodenafil, mirodenafil, and udenafil, some of which are commercially available in a few countries (lodenafil in Brazil; mirodenafil in South Korea; and udenafil in South Korea, Russia, and Philippines) [113]. Other agents with weak PDE5I properties include icariin and zaprinast [116]. Icariin, a prenylated flavonol glycoside extracted from plants of the *Epimedium* genus, has demonstrated PDE5I activity in vitro, enhancement of NO, and antioxidant activity [116]. It has been widely used in Chinese traditional medicine. It shows peak concentration levels at 1 h and should be avoided in patients with bleeding disorders, hypotension, arrhythmias, and hormone-sensitive cancers (breast, ovarian, or prostate). Zaprinast is an inhibitor of PDE5, PDE6, PDE9, and PDE11. In the past, it has been used for the treatment of PAH and inhibition of malaria parasites. Zaprinast activates the G-protein coupled receptor, GPR35, that plays a crucial role in cardiovascular disease, pain, regulation of inflammation, hypertension, diabetes, and irritable bowel disease [117,118]. The main characteristics of PDE5Is are summarized in Table 7 [34,112,113,119,120,121,122,123,124,125].

PDE5Is interfere selectively with cGMP hydrolysis by PDE5, increasing intracellular cGMP, which results in smooth muscle relaxation/raised arterial blood flow improving penile erection. PDE5 belongs to a superfamily of enzymes that convert intracellular cAMP/cGMP into the consonant nucleotides. It is a cytosolic protein with three isoforms expressed in various organs apart from the penis (corpora cavernosa), including kidney (vessels, glomeruli, inner medullary collecting ducts, and cortical tubules) that specifically degrades cGMP [66]. In particular, PDE5A1 and PDE5A2 are widely expressed in tubular epithelial cells of the renal proximal tubule and medullary collecting duct, as well as in vascular smooth muscle cells, platelets, brain, and lung, while PDE5A3 is only expressed in vascular smooth muscle cells [126].

Cyclic nucleotide signal transduction pathways represent an emerging research field in kidney disease, with selective PDE5 inhibition attracting ongoing interest nowadays [127]. Current evidence supports the notion that regulation of the cGMP -dependent protein kinase 1-PDE signaling pathway may be reno-protective and that its regulation might provide novel, therapeutic strategies for chronic kidney disease with selective PDE5Is having shown potential in treating kidney fibrosis, while possessing antithrombotic and anticancer activity [128]. In this respect, PDE5Is represent a potential but still understudied/controversial option against various forms of AKI such as CIN [28], given that NO/cGMP are crucial mediators in renal vasculature and NO is an essential endogenous vasodilator for medullary oxygenation [33].

**Figure 3:**
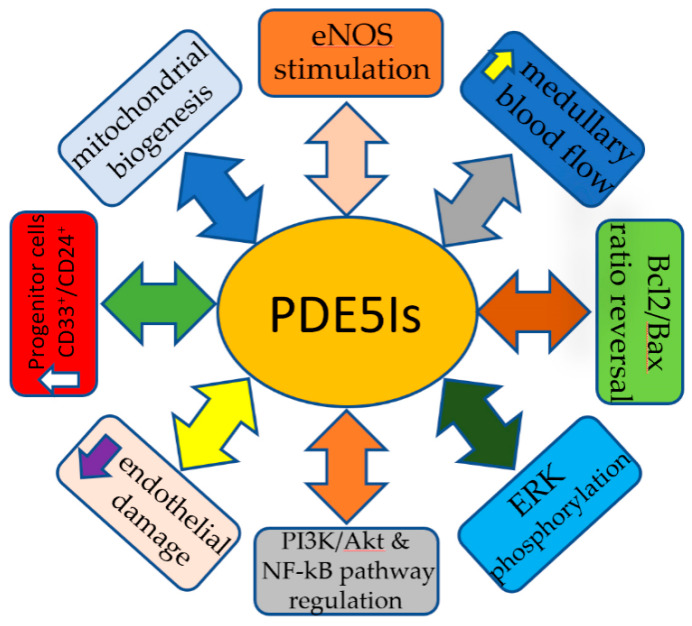
Reno-protective mechanisms of PDE5Is.

The mechanism of action of PDE5Is in the prevention and management of AKI is still not fully elucidated. Multiple mechanisms have been proposed to play a role in counteracting the cascade of changes caused by the renal injury. Stimulation of NO production through NOS, medullary blood flow improvement, protection against vascular endothelial damage, Bcl2/Bax ratio reversal, ERK phosphorylation, mitochondrial biogenesis activation, renal progenitor cell upregulation, and the regulation of multiple signaling pathways such as insulin/IGF1, T17/Treg, PI3K/Akt, and NF-kB [75] are the most well-described mechanisms through which PDE5Is offer protection. The increased ERK phosphorylation boosts NOS activity and subsequent rapid NO release [30]. The repairing process following any renal injury requires energy provided by the cellular mitochondria. Mitochondria are continuously regenerated but cellular injury such as sepsis and hypoxia induce rapid biogenesis. This process is mediated by a transcriptional co-activator, peroxisome proliferator-activated receptor γ co-activator 1a (PGC-1a). PGC-1a activates the nuclear respiratory factors 1 and 2, which eventually activate mitochondrial transcription factor A that is responsible for the transcription of mitochondrial DNA [23,67]. An alternative process that PDE5Is activate to promote recovery from renal injury is the renal progenitor cell stimulation. PDE5Is, more specifically icariin, upregulates HGF, WT-1, and BMP-7, which lead to an increased number of CD133^+^ and CD24^+^, cells that are capable of self-renew and also differentiate into podocytes or tubular cells [57,103]. In addition to the aforementioned actions, PDE5Is are likely to exert their protective effect through an alternative pathway. PDE5Is increase cGMP, which activates protein kinase G that opens mitochondrial K_ATP_ channels that induce depolarization of the mitochondrial inner membrane and Mg^2+^ release. The depolarized membrane results in reduced Ca^2+^ influx; therefore, suppressed cellular death and increased Mg^2+^ concentration reduces ROS and lessens p38 MAPK activation, which is responsible for apoptosis [30,88,129]. The most common reno-protective mechanisms of PDE5Is are summarized in Figure 3.

To the best of our knowledge, this is the first review that attempts in a systematic way to define the reno-protective potential of PDE5Is in the various forms of AKI. Based on our results, it appears that sildenafil is the most widely PDE5I studied in AKI among the 11 natural/synthetic agents currently available (avanafil, benzamidenafil, dasantafil, icariin, iodenafil, mirodenafil, sildenafil, tadalafil, udenafil, vardenafil, and zaprinast).

The reno-protective effects of PDE5Is have been evaluated in four human studies to date (preclinical studies using human cells: n = 2 [24,38]; clinical studies: n = 2 [17,39]) (Table 1). In one study, human umbilical cord mesenchymal stem cells (huMSC), which have a high self-renewal/multi-directional differentiation potential, were treated with icariin and administered in an animal model of renal injury induced by adenine [38]. Blood urea nitrogen/sCr analysis showed amelioration of functional parameters. Icariin-treated huMSC increased the number of cells in injured renal tissues, reduced fibrosis, oxidative damage, inflammatory responses, and promoted expression of growth factors protecting injured renal tissue. In another study, cisplatin was added to human embryonic kidney (HEK)-293 renal cell cultures pre-treated with icariin [24]. The authors concluded that icariin prevents cisplatin-induced HEK-293 cell injury by inhibiting oxidative stress, inflammatory response, and cellular apoptosis partly via regulating nuclear factor kappa-like chain-enhancer of activated B cells (NF-κB) and PI3K/Akt signaling pathways. In a non-randomized clinical trial, 49 patients with renal tumors were submitted to open nephron-sparing surgery after renal artery clamping [17]. Twenty-two patients were pre-treated with tadalafil one day pre- and two days post-operatively and 27 patients underwent the same surgery without receiving tadalafil. Renal artery clamping induced kidney dysfunction reflected by increases in urinary NGAL and KIM-1 (two novel biomarkers for AKI) in all participants. Tadalafil reduced the urinary excretion of KIM-1, but not of NGAL. The incidence of AKI was comparable between groups but sCr elevation was significantly attenuated in the tadalafil-treated group compared to controls. It was concluded that tadalafil exerts reno-protective effects in AKI following nephron-sparing surgery. In a randomized placebo-controlled trial, 40 patients were submitted to robot-assisted partial nephrectomy after hilar clamping. The reno-protective effect of a single 100 mg oral dose of sildenafil immediately prior to clamping was evaluated [39]. GFR was similarly decreased between arms during the immediate postoperative period and at an intermediate-term follow-up of six months; the reno-protective effect of sildenafil was not evident in this study (neutral effect).

All animal studies investigating the potential reno-protective effect of sildenafil (n = 41) manifested a beneficial effect, irrespectively of the mechanism of AKI; almost all parameters evaluated (biochemical or morphological) were reported to improve (Table 2 and Table A1). Similarly, all animal studies investigating the potential reno-protective effect of tadalafil (n = 19) revealed beneficial outcomes (attenuated histopathological changes/improved biochemical profile; Table 3 and Table A2). Two studies provided comparative results for sildenafil and tadalafil, demonstrating the superiority of the former against tubular cell apoptosis, oxidative status, lipid peroxidation and NOS alterations [45,49]. Unique proteins, cells, and genes have been utilized to investigate the aftermath following icariin’s administration as a reno-protective agent, such as connective tissue growth factor, TUNEL positive cells, nephrin, α-smooth muscle actin, E-cadherin, LY6G, F4/80, NLRP3, NF-κΒ, etc. All available animal studies evaluating icariin (n = 8) showed a beneficial effect (oxidative injury reversal, obliteration of renal function impairment, and improvement of renal hemodynamics/NO sensitivity; Table 4 and Table A3). Similar to sildenafil/tadalafil, icariin suspends the inflammatory response initiation as well as the alteration of the cellular phase and preserves renal morphology. Finally, vardenafil, zaprinast, and udenafil have been investigated in a limited number of studies (n = 7, n = 4, and n = 2, respectively), almost all of which show antioxidant, anti-inflammatory, and reno-protective effects (Table 5, Table 6, Table A4, and Table A5). In one study, vardenafil was compared to sildenafil and tadalafil in an animal model of partial unilateral ureteric obstruction, reporting that all agents were beneficial with sildenafil showing best results [45]. Only one study failed to demonstrate any beneficial effect from zaprinast pre-treatment in an animal model of nephrectomy and concomitant contra-lateral renal hilar occlusion [40]. Even though data are still limited, especially in humans with inconclusive or negative results of only two clinically relevant studies available at present, the results of animal studies are promising and have already fueled clinical research, which is on-going with results expected to come out in the near future [122]. Nevertheless, the potential reno-protective capacity of PDE5Is in AKI warrants further investigation in clinical trials.

## 5. Conclusions

PDE5Is appear to be beneficial in AKI of various etiologies by improving renal functional/histopathological alternations through various mechanisms, such as by affecting regional hemodynamics, cell expression, and mitochondrial response to oxidative stress and inflammation. The reno-protective action of PDE5Is was evident in the vast majority of the studies, independently of the AKI type and the agent applied. Even though data are still limited, especially in humans with inconclusive or negative results of only two clinically relevant studies available at present, the results of animal studies are promising. The potential reno-protective capacity of PDE5Is in AKI warrants further investigation in clinical trials.

## Figures and Tables

**Table 1:** Human studies evaluating the potential reno-protective effects of phosphodiesterase 5 inhibitors.

Reference Country/Year	Type of Study	AKI Model	PDE5I Route	Timing	Sample	AKI Renal Effects	PDE5I Renal Effects	Outcome
[24]/ China/2019	Preclinical study on HEK-293 cell culture	Cisplatin Various doses Finally chosen 20 μΜ dose 24 h	Icariin Various doses (0.25–2.0 μΜ) 24 h prior to cisplatin	PRE	Centrifuged at 4 °C, 10,000 g, for 20 min	Reduced viability, ↑p-NF-Kb ↓GSH concentration ↑MDA levels,↑Bax, ↓Bcl-2 ↑ROS generation, ↑Caspace 3 ↑iNOS/TNF-a/IL-1β Nuclear fragmentation and cellular condensation	Improved viability, ↓p-NF-kB ↑GSH concentration ↓MDA levels, ↓Bax, ↑Bcl-2 ↓ ROS generation, ↓Caspace 3 ↓iNOS/TNF-a/IL-1β Blunted apoptotic changes Antiapoptotic action (PI3K/Akt pathway)	POS
[38]/ China/2017	Preclinical study using huMSCs in adult male Wistar rats	2.5% Adenine Orally 4 weeks +4th generation huMSCs	Icariin huMSCs were pretreated with 100 uM ICA for 1 week	PRE	3, 7, 14 days after treatment	↑Urine outputm, ↑Urea, ↑Cr ↑Damage renal tissue, ↑TNF-a ↓SOD, ↑MDA, ↑IL-6, ↑IL-10	↓Urine output,↓Urea, ↓Cr ↓Damage renal tissue, ↓TNF-a ↑SOD, ↓MDA, ↓IL-6, ↓IL-10 ↑BMP-7, ↑bFGF	POS
[17]/ Israel/2015	Clinical trial (non-RCT)	PN with 20 min cold ischemia	Tadalafil *Orally: *20 mg/day 1 day pre-operatively and 2 days postoperatively	PRE and POST	Pre-op and at 1,3,8, 24, 48, 72 h post op	↑NGAL, ↑KIM-1,↑sCr, ↓GFR	Attenuated all studied parameters	POS
[39]/ USA/2016	Clinical trial (RCT)	RAPN	Sildenafil *Orally* 100 mg prior to RAPN	PRE		↓GFR	↓GFR (No improvement)	NEUT

Abbreviations: AKI, acute kidney injury; Bax, proapoptotic protein; Bcl-2, antiapoptotic gene; bFGF, basic fibroblast growth factor; BMP-7, bone morphogenetic protein-7; GSH, glutathione; HEK, human embryonic kidney cells; huMSCs, human umbilical cord mesenchymal stem cells; iNOS, inducible NOS; IL, interleukin; LY6G, MDA, malondialdehyde; NOX-4, NADPH oxidase 4; PDE5I, phosphodiesterase 5 inhibitor; p-NF-Kb, phosphorylation nuclear factor kappa-light-chain-enhancer of activated B cells; PN, partial nephrectomy; RAPN, Robot assisted partial nephrectomy; RCT, randomized controlled trial; ROS, reactive oxygen species; sCr, serum creatinine; SOD, superoxide dismutase; TNF-a, tumor necrosis factor a; ↓, reduced; ↑, increased.

**Table 2:** Animal studies evaluating the potential reno-protective effects of sildenafil.

Reference /Country/Year	Studied Animal	AKI Model	PDE5I Route	Timing	Sample	AKI Renal Effects	PDE5I Renal Effects	Outcome
[41]/ South Korea/2009	Male Sprague Dawley rats	Cisplatin Single intraperitoneal injection 5 mg/kg	Sildenafil *Intraperitoneal* 0.4 mg/kg Just after the injection of cisplatin	POST	Left nephrectomy 96 h post cisplatin	↑BUN, ↑sCr, ↑Bax/Bcl-2 ratio ↑Caspase 3 expression ↑TUNEL positive cells Loss of brush border Vacuolation/Desquamation	↓sCr, ↓Bax/Bcl-2 ratio ↓Caspase 3 expression ↓TUNEL positive cells ↑eNOS and iNOS Significantly attenuated renal changes	POS
[30]/ Korea/2009	Male Sprague Dawley rats	IR renal injury model	Sildenafil *Intraperitoneal* 0.5 mg/kg 1 h prior to ischemia	PRE	Depending on the group 0-168 h after reperfusion	↑BUN, ↑sCr, ↑cGMP ↑Bax/Bcl-2 ratio, ↑Caspase 3 activity ↑TUNEL positive cells Loss of brush border Vacuolation/Desquamation	↓BUN, ↓sCr, ↑↑ cGMP ↓Bax/Bcl-2 ratio, ↓Caspase 3 activity ↓TUNEL positive cells ↑↑ ERK activity Attenuated all histological changes	POS
[42]/ Turkey/2010	Male Wistar albino rats	IR renal injury model	Sildenafil *Orally* 60 min pre-operatively	PRE	Left nephrectomy either at 45 min post occlusion or at 105 min post occlusion and reperfusion injury	↑MPO enzyme level and activity ↑TBARS Sclerosis of glomeruli Enlargement of Bowman space Loss of microvilli/Pyknotic nuclei Tubular necrosis/Interstitial edema Leucocyte infiltration Glomerular and tubular degeneration	↓MPO enzyme level and activity ⇔TBARS Attenuated tubular damage Preserved normal morphology Significantly decreased neutrophil infiltration	POS
[43]/ Brazil/2010	Wistar rats	IR renal injury model	Sildenafil *Orally* 1 mg/kg 60 min prior to ischemia	PRE	At 24 h and 7 days scintigraphy and nephrectomy	Scintigraphy: functional deficit representing ATN No PDE5i: ↑ cellular necrosis Vacuolation Intratubular cast formation	Reversed effect to normal split function PDE5i: just dilatation of tubular lumen No significant change in histology	POS
[44]/ Oman/2011	Male Wistar rats	Cisplatin Single intraperitoneal injection 5 mg/kg	Sildenafil *Intraperitoneally* 0.4 mg/kg for 5 days or Sildenafil *Subcutaneously* 10 mg/kg for 5 days	POST	Blood samples and bilateral nephrectomy 5 days post treatment	↓RBF, ↓BP, ↓Body weight ↑Urine output ↑BUN, ↑sCr, ↓CrCl ↑N-acetyl-β-D-glycosaminidase ↑TNF-a (plasma and renal) ↑Renal platinum concentration Acute Tubular Necrosis/Apoptotic cells	↑RBF, ↑BP (i.p.) No improvement in b.w. and u.o. ↓BUN, ↓sCr, ↑CrCl (i.p.) ↓N-acetyl-β-D-glycosaminidase Minimal improvement in TNF-a No change in platinum concentration Improvement of histological changes	POS
[45]/ Turkey/2011	Wistar albino rats	UUO model	Sildenafil-*orally*-1 mg/day Vardenafil-*orally*-0.5 mg/day Tadalafil-*orally*-10 mg/72 h For 30 days	POST	30 days	↑Tubular cell apoptosis ↑ eNOS ↑ iNOS	↓ Tubular cell apoptosis ↓ eNOS ↓ iNOS Sildenafil better results	POS
[46]/ Spain/2011	Minipigs	IR renal injury model	Sildenafil *Intravenously* 0.7 or 1.4 mg/kg 30 min prior to or during warm ischemia	PRE OR SIM	Monitoring of hemodynamics up to 45 min following unclamping		↓Systemic MAP (especially 1.4 mg/kg) ↑RVF (0.7 mg/kg)	POS
[47]/ Turkey/2011	Male Wistar rats	CLP model	Sildenafil Orally 10 or 20 mg/kg After the procedure	POST	16 h after CLP	↓SOD, ↓GSH, ↑MPO, ↑LPO ↑Mean inflammation score ↑TNF-a	↑SOD, ↑GSH, ↓MPO, ↓LPO ↓Mean inflammation score ↓TNF-a	POS
[48]/ United Kingdom	Female Large White Landrace crossbred pigs	Cardiopulmonary bypass 2.5 h	Sildenafil *Intravenously* 10 mg in 50 mL saline 0.9%	SIM	90 min pre-op 90 min post-op 24 h post-op	↓CCl, ↑Proteinuria, ↑IL-18 ↓ NO Pseudodilation of proximal tubules ↑iNOS ↑ cortical expression endothelin-1 Inflammatory cell infiltration	↑CCl ↓Proteinuria ↓IL-18 Significantly increased RBF (24 h) ↑NO Prevented phenotypic changes in proximal tubular cells ↓cortical expression endothelin-1 Preserved eNOS ↓iNOS ↓ inflammatory cell infiltration	POS
[49]/ Turkey/2012	Male Sprague Dawley rats	IR renal injury model	Sildenafil *Orally:* 1 mg/kg 60 min pre-operatively Tadalafil *Orally:* 1 mg/kg 60 min pre-operatively	PRE	Nephrectomy post procedure	↑MPO levels ↑MDA levels ↑iNOS gen, ↑eNOS expression ↑ apoptotic cells ↑p53 positive cells Leucocyte migration Edema/Tubular dilatation	MPO: no significant improvement ↓MDA (Sdf), ⇔ MDA (Tdf) levels ↓iNOS gen, ↓eNOS expression ↓apoptotic cell death ( Sdf > Tdf) ↓p53 positive cells All changes were attenuated	POS
[50]/ Germany/2013	NO-GC1 KO mice C57Bl/6Rj	UUO model	Sildenafil *Orally* 100 mg/kg In the 4th week post op	POST	4 weeks post op	↓cGMP ↓NO-stimulated guanyle cyclase activity (KO mice)	↑cGMP ↑NO sensitivity ↓SBP (more efficient in operated group rather than KO group)	POS
[23]/ USA/2013	Female New Zealand white rabbits	Folic Acid Intraperitoneally Single dose 250 mg/kg	Sildenafil *Intraperitoneally* 24 h after injury 0.3 mg/kg/day For 6 days	POST	Blood samples and kidneys were harvested 24 h post treatment	↓mRNA expression COX1 and Tfam ↓mtDNA copy number ↑KIM-1	↑mRNA expression COX1 and Tfam ↑mtDNA copy number ↓KIM-1	POS
[51]/ Egypt/2014	Sprague Dawley male rats	Cisplatin Intraperitoneally 6 mg/kg	Sildenafil *Intraperitoneally* 2 mg/kg 1 h before and 24 h after cisplatin injection	PRE and POST	96 h after cisplatin injection	↑BUN, ↑sCr, ↑MDA, ↑TNF-a ↑Caspase-3, ↓SOD ↓Nitrite/nitrate level Acute tubular necrosis	↓BUN, ↓sCr, ↓MDA, ↓TNF-a ↓Caspase-3, ↑SOD ↑Nitrite/nitrate	POS
[52]/ Turkey/2014	Adult female Wistar albino rats	Burn model	Sildenafil *Orally* 10 or 20 mg/kg just after burn	POST	24 h after the scald burn	Renal: ↑MDA, ↓Gpx, ↑VEGF ⇔ Flt-1, ⇔TAC, ⇔OSI, ⇔TOS Serum: ↑MDA, ↓Gpx, ⇔VEGF, ⇔Flt-1, ↓TAC, ⇔OSI, ↑TOS, ⇔Flt-1/VEGF ratio	Renal: ↓MDA, ↑Gpx, ↓VEGF ⇔Flt-1 (T10), ⇔TAC, ⇔OSI, ⇔TOS(T20) Serum: ↓MDA, ↑Gpx, ⇔VEGF ⇔Flt-1, ↑TAC, ↓OSI (T10) ↑Flt-1/VEGF ratio (T10) ↓TOS (T10) ↓Histopathological scores (no significant difference in T20)	POS
[53]/ Egypt/2014	Male Wistar rats	Gentamicin Intraperitoneally 100 mg/kg/day for 6 days	Sildenafil *Orally* 5 mg/kg/day for 6 days 1 h before gentamycin	PRE	24 h after last gentamycin injection	↑Cr, ↑Urea, ↑urinary albumin ↑MDA, ↑nitrite/nitrate levels ↓CAT (renal), ↓SOD, ↑iNOS, ↓eNOS Degeneration and necrobiosis in epithelial cells	↓Cr, ↓Urea, ↓urinary albumin ↓MDA, ↓nitrite/nitrate levels ↑CAT (renal), ↑SOD ↓iNOS, ↑eNOS Reversed histological alterations	POS
[54]/ USA/2014	Male wild-type (WT) littermates or PKG Tg mice	UUO model	Sildenafil *Subcutaneously* 12 mg/kg twice daily for 14 days	POST	14 days	↓Renal PKG activity Increase (↑) at Ang II, Collagen type I, III mRNA, α-SMA, E-cadherin, TNF-a, TGF-β1, pSmad2, ICAM-1 ↑Macrophage infiltration	↑Renal PKG activity Decrease (↓) at Ang II, Collagen type I, III mRNA, α-SMA, E-cadherin, TNF-a, TGF-β1, pSmad2, ICAM-1 ↓Macrophage infiltration	POS
[55]/ Brazil/2014	New Zealand white rabbits	CIN model	Sildenafil *Orally* 6 mg/kg before CM or 6 mg/kg before CM and 8 hourly for 48 h	PRE and POST	1/2/24/48 h	No changes in kidney to body weight ratio ↑sCr ↓Na, ↑K Multifocal tubular necrosis Tubular degeneration Luminal protein casts	No significant changes in kidney to body weight ratio ↓↓sCr (continuous) ↑Na, ↓K Continuous treatment blunted all changes	POS
[56]/ Egypt/2015	Male Sprague-Dawley rats	IR renal injury model	Sildenafil *Orally* (1 mg/kg) 60 min before anesthesia	PRE	Blood + urine samples (basal, at 2, 24, 48 h and 7 days) + Kidney tissue	↑sCr, ↑BUN, ↓Bcl-2 ↓Nrf2/HO-1/NQO-1 (genes) ↑ Proinflammatory cytokine genes (TNF-a, ICAM-1, IL-β) ↓Nrf 2 protein expression Acute tubular necrosis, detachment of epithelial cells from basement membrane, intracellular cast formation, loss of brush border, neutrophil infiltration	No improvement in BUN/sCr, ↑Bcl-2 ↑Nrf2/HO-1/NQO-1 (genes) ↓ Proinflamamtory cytokine genes (TNF-a, ICAM-1, IL-β) ↑ Nrf 2 protein expression Improved histological features of renal injury (mild tubular necrosis)	POS
[57]/ Brazil/2016	Male Wistar rats	CIN model	Sildenafil *Orally* 50 mg/kg/d 7 days (started 5 days before CM)	PRE and POST	48 h after CM administration	↑BUN, ↑sCr, ↑urine protein ↓GFR, ↓RPF, ↑RVR ↑superoxide anions production ↑H_2_O_2_ production ↑peroxynitrite and hydroxyl production ⇔ NO Reduced body weight Renal hypertrophy	↓BUN, ↓sCr, ↓urine protein ↑GFR, ↑RPF, ↓RVR ⇔superoxide anions production ↓ H_2_O_2_ production ↓peroxynitriteand hydroxyl production ⇔ NO No effect of PDE5 on histological changes	POS
[58]/ Egypt/2016	Male Wistar albino rats	IR renal injury model	Sildenafil *Intraperitoneally* (0.5 + 1.0 mg/kg) 1 h before ischemia	PRE	Blood/kidney tissue samples 24 h after reperfusion	↓CrCl, ↑ BUN, ↑Uric acid, ↑FeNa ↑Plasma potassium ↓GSH levels,↑TBARS, ↑SAG levels Glomerular damage, detachment of basement membrane, loss of brush border, tubular dilation, atroprhy, neutrophil accumulation	↑CrCl, ↓BUN, ↓Uric acid ↓FeNa ↓Plasma potassium ↑GSH levels, ↓TBARS ↓ SAG levels ↓Renal tissue damage	POS
[59]/ Turkey/2018	Female Wistar albino rats	CIN model	Sildenafil *Orally* 50 mg/kg 48 h prior to CM	PRE	48 h after CM administration	↑HIF-2a (serum and tissue) ↑ BUN, ↑Cr (serum and urine) Hemorrhage, shedding of brush border, tubular vacuolization, degeneration, inflammatory cell infiltration, intratubulat cast obstruction	↓HIF-2a (serum and tissue) ↓ sCr Sildenafil improved all histological changes	POS
[60]/ Egypt/2018	Male albino rats	Cisplatin 5 mg/kg Single dose intraperitoneally	Combination Sildenafil, *Orally* 40 mg/kg Gemfibrozil-*Orally*– 100 mg/kg 14 days prior or after	PRE OR POST	Day 17	↑sCr, ↓HO-1, ↓GSH ↓eNOS, ↓TNF-a ↑Tubular injury/tubular necrosis	All changes improved with sildenafil and gemfibrozil especially in the group given after cisplatin	POS
[61]/ Egypt/2019	Mongrel dogs (aged 2-3 years)	IR renal injury model	Sildenafil *Orally* 1 mg/kg 1 h prior to operation or *In the perfusion fluid* 0.5 mg/kg during the operation	PRE OR SIM	Prior and at the end of the experiment (Day 1,3,7,14)	↑sCr, ↑BUN, ↓GFR ↑caspase 3, ↑Nrf2 ↑TNF-a, ↑ IL-1Β, ↑ICAM -1 ↓eNOS Renal degeneration Cortical and medullary interstitial fibrosis	↓sCr, ↓BUN, ↑GFR ↓caspase 3, ↑↑Nrf2 ↓TNF-a, ↓IL-1Β, ↓ICAM -1 ↑eNOS Significantly improved all histological changes	POS

Abbreviations: AKI, acute kidney injury; Ang II, angiotensin II; Bax, proapoptotic protein; Bcl-2, antiapoptotic gene; BP, blood pressure; BUN, blood urea nitrogen; Ca^2+^, calcium; CAT, catalase; cGMP, cyclic guanosine monophosphate; CIN, contrast induced nephropathy; CLP, caecal ligation and puncture; COX, cyclo-oxygenase CrCl, creatinine clearance, eNOS, endothelial NOS, FeNa, fractional excretion of sodium, GFR, glomerular filtration rate; GPx, glutathione peroxidase; GSH, glutathione; HIF-2a, heterodimeric nuclear transcription factor-2 alpha; HO-1, heme oxygenase 1; IR, ischemia reperfusion; ICAM-1, intercellular adhesion molecule 1; IL, interleukin; iNOS, inducible NOS; K, potassium; KIM-1, kidney injury molecule-1; LPO, lipid peroxidation; MAP, mean arterial pressure; MDA, malondialdehyde; MPO, myeloperoxidase; Na, sodium; NO, nitric oxide; NRF2, nuclear erythroid related factor 2; OSI, oxidative stress index; P, phosphorus; PDE5I, phosphodiesterase 5 inhibitor; PKG, protein kinase G; pSmad2, antibody; RBF, renal blood flow; RPF, renal plasma flow; RRI, renal resistive index; RVF, renal vascular flow; RVR, renal vascular resistance; SAG, superoxide anion generation; sCr, serum creatinine; sFlt1, soluble fms-like tyrosine kinase-1; SOD, superoxide dismutase; SBP, systolic blood pressure; TAC, total antioxidant capacity; Tfam, mitochondrial transcription factor; TGF-β1, transforming growth factor beta 1; TBARS, thiobarbituric acid reactive substances; TNF-a, tumor necrosis factor a; TOS, total oxidant status; TUNEL, terminal deoxynucleotidyl transferase dUTP nick end labeling; UUO, unilateral ureteral obstruction; VEGF, vascular endothelia growth factor; ↓, reduced; ↑, increased ⇔, no change.

**Table 3:** Animal studies evaluating the potential reno-protective effects of tadalafil.

Reference/ Country/Year	Studied Animal	AKI Model	PDE5I Route	Timing	Sample	AKI Renal Effects	PDE5I Renal Effects	Outcome
[62]/ Turkey/2019	New Zealand rabbits	UUO model	Tadalafil *Orally* 10 mg/72 h for 30 days prior to obstruction	PRE	4th hour and 1^st^ and 3rd day	↑Resistivity index ↑Pulsatility index	↓Resistivity index ↓Pulsatility index In the non-obstructed kidney reduced resistivity index at 4th hour then normal	POS
[63]/ Turkey/2011	Male Sprague Dawley rats	IR renal injury model	Tadalafil *Orally* 1 mg/kg 60 min pre-operatively	PRE	At 45 min post occlusion or at 105 min post occlusion and reperfusion injury	Sclerosis of glomeruli Enlargement of Bowman space Loss of microvilli/Tubular necrosis Interstitial edema/Leucocyte infiltration Hyaline degeneration	Attenuated histological changes and decreased neutrophil infiltration	POS
[45]/ Turkey/2011	Wistar albino rats	UUO model	Sildenafil-*orally*-1 mg/day Vardenafil-*orally*-0.5 mg/day Tadalafil-*orally*-10 mg/72 h For 30 days	POST	30 days	↑Tubular cell apoptosis ↑eNOS ↑iNOS	↓Tubular cell apoptosis ↓eNOS ↓iNOS Sildenafil better results	POS
[64]/ Turkey/2011	Male Wistar albino rats	IR renal injury model	Tadalafil *Orally* 10 mg/kg 60 min pre-operatively	PRE	Left nephrectomy at 120 min post-operatively	↑Total oxidant status Tubular necrosis/Vacuolization Congestion/Mononuclear cell infiltration	↑ Total antioxidant status Reduced all injuries to the renal tissue.	POS
[65]/ USA/2012	Adult female pigs	IR renal injury model	Tadalafil 40 mg Two doses (12 h before and just prior to surgery)	PRE	Induction and Days 1, 3, 7 post occlusion	↑Creatinine after nephrectomy ↑↑ Creatinine Day 1 post ischemia	↓Creatinine after nephrectomy No significant change in creatinine Day 1 post ischemia	POS
[49]/ Turkey/2012	Male Sprague Dawley rats	IR renal injury model	Sildenafil *Orally:* 1 mg/kg 60 min pre-operatively Tadalafil *Orally:* 1 mg/kg 60 min pre-operatively	PRE	Nephrectomy post procedure	↑ MPO levels ↑MDA levels ↑iNOS gen, ↑eNOS expression ↑apoptotic cells ↑p53 positive cells Leucocyte migration Edema/Tubular dilatation	MPO: no significant improvement ↓MDA (Sdf), ⇔MDA (Tdf) levels ↓iNOS gen, ↓eNOS expression ↓apoptotic cell death (Sdf > Tdf) ↓p53 positive cells All changes were attenuated	POS
[66]/ Israel/2013	Male Sprague Dawley rats	IR renal injury model	Tadalafil *Orally* 10 mg/kg 24-hr prior to ischemia	PRE	30/60 min after nephrectomy 60/120/180/240 min after clamping	↑V, ↑U_Na_V, ↑FeNa, ↓GFR, ⇔RPF, ↑NGAL, ↑KIM-1 Tubular dilatation/Loss of brush border Necrosis and cast formation	↓V, ↓U_Na_V, ↓FeNa, ↑GFR, ↑RPF, ↓NGAL, ↓KIM-1 Blunted all changes	POS
[67]/ China/2014	Male Wistar rats	Sepsis model	Tadalafil *Orally* 10 mg/kg 24 h prior to procedure for 28 days	PRE and POST	Nephrectomy and samples at: 8 days post treatment and 6 weeks post treatment	↑Systolic and diastolic BP, ↑NO ↑BUN, ↑sCr, ↑MDA levels ↓SOD, ↑TGF-β	↓Systolic and diastolic BP, ↓NO, ↓BUN, ↓sCr, ↓MDA levels, ↑SOD ↑IL-10, ↓TNF-a, ↓IL-1β, ↓TGF-β ↓RANTES, ↓MIP-1β, ↓MCP-1	POS
[68]/ Turkey/2015	Female Wistar albino rats	IR renal injury model	Tadalafil Orally 10 mg/kg 24 h prior to procedure	PRE	Cardiac blood samples and nephrectomy after reperfusion injury	No significant difference Severe tubular dilatation degeneration and necrosis/Enlargement of Bowman capsule	in IMA/NO/MDA levels Blunted all changes	POS
[69]/Turkey/2015	Wistar albino rats	IR renal injury model	Tadalafil *Intraperitoneally* 10 mg/kg Immediately prior to procedure	PRE	Blood samples and nephrectomy following 60 min of reperfusion injury	↑MDA levels (serum/renal) ↓TAC levels (serum/renal) ↑APAF-1, ↑iNOS, ↑eNOS Loss of nucleus/Cellular edema Vacuolization/Brush border loss Tubular dilatation/edema Interstitial congestion	⇔MDA (renal), ↓MDA (serum) ⇔TAC (renal), ↑TAC (serum) ↓APAF-1, ↓iNOS, ↓eNOS Damage was significantly less after tadalafil treatment	POS
[35]/Turkey/2015	Female Wistar albino rats	CIN model	Tadalafil *Orally* 10 mg/kg immediately after contrast	POST	48 h after CM administration	Significant weight loss after dehydration ↑Serum cystatin C ↑BUN, ↑sCr, ↑MDA Medullary congestion	Significant weight loss after dehydration ↓Serum cystatin C ↓BUN, ↓sCr, ↓MDA Similar histological findings	POS
[29]/ Egypt/2016	Adult male albino rats	IR renal injury model	Tadalafil *Orally* (5 mg/kg) Pre-treatment	PRE	Blood/kidney tissue samples 6 h after reperfusion	↑sCr, ↑BUΝ, ↑MDA levels ↓SOD activity, ↑MPO activity ↑ICAM-1, ↑TNF-a, ↑IL-1β ↑Caspase-3 activity Congestion and interstitial hemorrhage, proximal and tubular necrosis	↓sCr, ↓BUΝ, ↓MDA levels ↑SOD activity, ↓MPO activity ↓ICAM-1, ↓TNF-a, ↓IL-1β ↓Caspase-3 activity Dilated proximal, distal, and collecting tubules and interstitial connection	POS
[70]/ Nigeria/2016	Male Wistar rats	Cisplatin Intraperitoneal 5 mg/kg	Tadalafil *Orally:* 2 or 5 mg/kg for 7 days pretreatment	PRE	Blood samples and renal tissue obtained 3 days post cisplatin	↓Na/K/HCO_3/_Ca^2+^/P ↑BUN, ↑sCr, ↑MDA/GPx ↓GSH/SOD/CAT (renal)	Significant attenuation of all histological and biochemical alterations	POS
[71]/ Israel/2017	Male albino Wistar rats	CLP model	Tadalafil *Orally* 5 or 10 mg/kg End of the procedure	POST	Left nephrectomy + Blood samples 16 h postoperatively	↓CAT, ↓SOD, ↑IL-6, ↑sCr, ↑MPO, ↑MDA, ↑Cystatin C ↑Mac387 antibody ↑Tubular injury, glomerulus deformities ↑Inflammatory cell infiltration	↑CAT, ↑SOD, ↓IL-6, ↓sCr, ↓MPO, ↓MDA, ↓Cystatin C ↓Mac387 antibody ↓Tubular injury, glomerulus deformities ↓Inflammatory cell infiltration	POS
[72]/ Brazil/2017	Male Wistar rats	IR renal injury model	Tadalafil *Orally* 10 mg/kg 1 h pre-procedure	PRE	After nephrectomy	Interstitial Leucocyte accumulation	Successful reversal by tadalafil	POS
[73]/ Brazil/2017	Male Wistar rats	IR renal injury model	Tadalafil *Orally:* 10 mg/kg 1 h before ischemia	PRE	Fluorescence imaging (ICG) Blood samples	↓ICG signal, ↑TNF-a, ↑IL-1β ↑IL-6 ↑BUN, ↑sCr, ↑CRP	↑ICG signal, ↓TNF-a, ↓IL-1β ↓IL-6 ↓BUN, ↓sCr, ↓CRP	POS
[74]/ Turkey/2019	Male Sprague Dawley rats	UUO model	Tadalafil *Orally* 10 mg/72 h	---	15 days post ligation	↑aSMA, ↑TGF-β Partial: inflammatory cell infiltration/severe epithelial atrophy/edema of epithelial cells/vacuolation Complete: macrophage infiltration/hemorrhage/irregular dark nuclei/thinner epithelium/denuded epithelial cells	↓aSMA, ↓TGF-β Attenuation of all changes with tadalafil	POS

Abbreviation: AKI, acute kidney injury; APAF-1, apoptotic protease activating factor 1; aSMA, α-smooth muscle actin; BUN, blood urea nitrogen; Ca^2+^, calcium; CAT, catalase; CIN, contrast induced nephropathy; CLP, caecal ligation and puncture; CRP, c-reactive protein; eNOS, endothelial NOS; FeNa, fractional excretion of sodium; GFR, glomerular filtration rate; GPx, glutathione peroxidase; GSH, glutathione; HCO_3_^−^, bicarbonate; IR, ischemia/reperfusion; ICAM-1, intercellular adhesion molecule 1; IL, interleukin; ICG, indocyanine green; IMA, ischemia modified albumin; iNOS, inducible NOS; K, potassium; KIM-1, kidney injury molecule-1; Mac387, Macrophage antibody; MCP-1, monocyte chemoattractant protein 1; MDA, malondialdehyde; MIP-1β, macrophage inflammatory protein-1β; MPO, myeloperoxidase; Na, sodium; NGAL, neutrophil gelatinase-associated lipocalin; NO, nitric oxide; P, phosphorus; PDE5I, phosphodiesterase 5 inhibitor; RANTES, Regulated upon Activation Normal T-cell Expressed, and Secreted; RPF, renal plasma flow; sCr, serum creatinine; Sdf, sildenafil; SOD, superoxide dismutase; TAC, total antioxidant capacity; Tdf, tadalafil; TGF-β1, transforming growth factor beta 1; TNF-a, tumor necrosis factor a; UNaV, urine sodium volume; UUO, unilateral ureteral obstruction; V, urine volume; ↓, reduced; ↑, increased ⇔, no change.

**Table 4:** Animal studies evaluating the potential reno-protective effects of icariin.

Reference/ Country/Year	Studied Animal	AKI Model	PDE5I Route	Timing	Sample	AKI Renal Effects	PDE5I Renal Effects	Outcome
[75]/ China/2015	Male BALB/c mice	Cisplatin 15 mg/kg Intraperitoneal	Icariin *Orally* 30 or 60 mg/kg/day For 6 days	PRE	At 6 days	↑BUN, ↑sCr, ↑MDA ↓GSH concentration, ↓Catalase ↓SOD activity, ↑TNF-a, ↑NF-Kb ↑TUNEL positive cells ↑Caspase-3, ↓Bcl-2 Tubular congestion/edema Loss of brush border/Tubular cell flattening and necrosis/nuclear pyknosis Severe invasion of inflammatory cells	↓BUN, ↓sCr,↓MDA ↑GSH concentration, ↑Catalase ↑SOD activity, ↓TNF-a, ↓NF-kB ↓TUNEL positive cells ↓Caspase-3, ↑Bcl-2 Partial improvement of the features (dose dependent)	POS
[18]/ China/2018	Male C57BL/6N mice	CLP model	Icariin *Orally* 30 or 60 mg/kg 3 days prior to surgery	PRE	Observed for 5 days	↑BUN, ↑sCr, ↑MDA levels ↑IL-1β/IL-6/TNF-a ↑ NF-κB ↓ GSH concentration ↓Catalase, ↓SOD activity ↑TUNEL +ve cells ↑Renal vascular permeability ↑Bax,↓Bcl-2, ↑Caspase 3 Extensive tubular necrosis/Loss of brush border	↓BUN, ↓sCr, ↓MDA levels ↓IL-1β/IL-6/TNF-a, ↓ NF-κB ↑GSH concentration ↑Catalase, ↑SOD activity ↓TUNEL +ve cells (60>30) ↓Renal vascular permeability ↓Bax, ↑Bcl-2, ↓Caspase 3 ↑Survival (both doses) Improvement in all histological features	POS
[76]/ Taiwan/2019	Adult C57BL/6J	UUO model	Icariin *Orally* 20 mg/kg/day For 3 days prior and 3, 7, or 14 days after	PRE and POST	3, 7, or 14 days post ligation	↑TGF-β, ↑α-SMA ↑fibronectin ↑NOX-4, ↓E-cadherin, ↓SOD-1 ↓Catalase, ↑CTGF, ↑Ly6G ↑F4/80, ↑phosphorylation IL-1β ↑Phosphorylation COX-2/NF-κΒ-65 Tubular dilatation/interstitial cell proliferation/inflammatory cell infiltration/tuft to capsule glomerular adhesions/collagen deposition	↓TGF-β, ↓α-SMA, ↓fibronectin ↓NOX-4,↑E-cadherin, ↑SOD-1 ↑Catalase, ↓CTGF, ↓Ly6G ↓F4/80, ↓phosphorylation IL-1β ↓Phosphorylation COX-2/NF-κΒ-65 Non-significant moderate reversal by icariin	POS

Abbreviations: AKI, acute kidney injury; Bcl-2, antiapoptotic gene; BUN, blood urea nitrogen; CLP, caecal ligation and puncture; COX, cyclo-oxygenase; CTGF, connective tissue growth factor; F4/80, macrophage marker; GSH, glutathione; IL, interleukin; LY6G, neutrophil marker; MDA, malondialdehyde; NF-κB, nuclear factor kappa-like chain-enhancer of activated B cells; NOX-4, NADPH oxidase 4; PDE5I, phosphodiesterase 5 inhibitor; sCr, serum creatinine; SOD, superoxide dismutase; TGF-β1, transforming growth factor beta 1; TNF-a, tumor necrosis factor a; TUNEL, Terminal deoxynucleotidyl transferase dUTP nick end labeling; UUO, unilateral ureteral obstruction; ↓, reduced; ↑, increased.

**Table 5:** Animal studies evaluating the potential reno-protective effects of vardenafil.

Reference/ Country/Year	Studied Animal	AKI Model	PDE5I Route	Timing	Sample	AKI Renal Effects	PDE5I Renal Effects	Outcome
[45]/ Turkey/2011	Wistar albino rats	UUO model	Sildenafil-*orally*-1 mg/day Vardenafil-*orally*-0.5 mg/day Tadalafil-*orally*-10 mg/72 h For 30 days	POST	30 days	↑Tubular cell apoptosis ↑eNOS ↑ iNOS	↓Tubular cell apoptosis ↓ eNOS ↓iNOS Sildenafil better results	POS
[77]/ Greece/2013	Male Wistar rats	IR renal injury model	Vardenafil *Intravenously* 0.02, 0.2, 2, 20 μg/kg 1 h pre-operatively or 2μg/kg 45 min post occlusion	PRE or POST	Blood samples and right nephrectomy 4 h post ischemia	Edema Loss of brush border Nuclear condensation	↓sCr (0.2, 2, 20 μg/kg) No change when given post-ischemia ↓FENa, ↑Renal uptake of tracer ↑cGMP, ↑ERK 1/2 phosphorylation Renoprotection (in scintigraphy) Significant improvement in all histo-logical changes irrespectively of dose	POS
[78]/ Brazil/2015	Male Wistar rats	IR renal injury model	Vardenafil *Solution in a probe* (1 mg/mL in 10 mg/kg) 1 h prior the ligation	PRE	Left nephrectomy Cytophotometry 24 h after reperfusion	↑Cleaved caspase-3 ↑sCr ↑Vacuolar degeneration	↓ Cleaved caspase-3 ↓ Vacuolar degeneration	POS

Abbreviations: AKI, acute kidney injury; cGMP, cyclic guanosine monophosphate; eNOS, endothelial NOS; ERK, extracellular signal-regulated kinase; FeNa, fractional excretion of sodium; IR, ischemia/reperfusion; iNOS, inducible NOS; PDE5I, phosphodiesterase 5 inhibitor; sCr, serum creatinine; UUO, unilateral ureteral obstruction; ↓, reduced; ↑, increased.

**Table 6:** Animal studies evaluating the potential reno-protective effects of zaprinast and udenafil.

Reference/ Country/Year	Studied Animal	AKI Model	PDE5I Route	Timing	Sample	AKI Renal Effects	PDE5I Renal Effects	Outcome
[79]/ USA/1995	Male Sprague-Dawley rats	IR renal injury model	Zaprinast *Intravenously* 0.03 and 0.3 mg/kg/min 24 h after ischemia	POST	During clamping, PDE5i infusion, up to 6 days following ischemia	↑sCr, ↓GFR	↓sCr, ↑GFR, ↓Low MAP ↑U_Na_V, ↑Urinary cGMP ↑Cortical and medullary blood flow	POS
[40]/ USA/2013	Female Sprague-Dawley rats	IR renal injury model	Zaprinast *Intraperitoneally* 10 mg/kg or 20 mg/kg Single dose 30 min pre-operatively	PRE	24 h post operatively blood samples and left nephrectomy		No statistically significant differrences in either BUN levels or sCr levels or histologic scores or TUNEL positive cells	NEUT
[80]/ Germany/2017	6-8-week-old mice	UUO model	Zaprinast, *Intraperitoneally* 10 mg/kg/day for 7 days	POST	After 7 days	↑cGMP, ↑sCr	↑↑cGMP, ↑MMP9, ↑TGF-β ⇔sCr, ↓Collagen	POS
[21]/Turkey/2017	Female Wistar albino rats	IR renal injury model	Udenafil *Orally:* 10 mg/kg 1 h prior to clamping	PRE	60 min and 24 h after reperfusion	↑BUN, ↑sCr ↑MDA, ↑NGAL	↓BUN, ↓sCr ↓MDA, ↓NGAL Lowest pathological damage rates	POS

Abbreviations: AKI, acute kidney injury; BUN, blood urea nitrogen; cGMP, cyclic guanosine monophosphate; GFR, glomerular filtration rate; IR renal, ischemia/reperfusion; MAP, mean arterial pressure; MDA, malondialdehyde; MMP9, Matrix metallopeptidase 9; NGAL, neutrophil gelatinase-associated lipocalin; PDE5I, phosphodiesterase 5 inhibitor; sCr, serum creatinine; TUNEL, terminal deoxynucleotidyl transferase dUTP nick end labeling; UNaV, urinary sodium excretion; UUO, unilateral ureteral obstruction; ↓, reduced; ↑, increased ⇔, no change.

**Table 7:** Main characteristics of phosphodiesterase 5 inhibitors.

PDE5i	FDA Approved	Launch Date	Pharmacokinetics	Recommended Dosage	Indications	Side Effects	Contraindications	Emerging and Other Off-Label Therapeutic Applications
Sildenafil	**Yes**	1998	Cmax = 560 µg/L Tmax = 0.8–1 h T1/2 = 2.6–3.7 h Affected by heavy/fatty meals	ED: 25–100 mg OD PAH: 5–20 mg TDS	ED PAH	Headache: 12.8% Flushing: 10.4% Dyspepsia: 4.6% Nasal congestion: 1.1% Dizziness: 1.2% Abnormal vision: 1.9%	Absolute: Any form of organic nitrate or NO donors Myocardial infarction, stroke, or life-threatening arrhythmia within the last 6 months Resting BP <90/50 or >170/100 Unstable angina, angina with intercourse, CHF NYHA IV Relative: Known serious hypersensitivity reaction Antihypertensive medication a-blockers Drugs that inhibit CYP34A	Penile rehabilitation after Radical Prostatectomy Heart Failure/CVD High altitude illness Stroke/Neurodegenerative diseases Peripheral neuropathy Improving fertility Peripheral Arterial Disease Raynaud’s syndrome Diabetic Nephropathy AKI CKD Stuttering priapism Premature ejaculation Ureteral stones Reyronie’s disease Female sexual dysfunction Overactive bladder Diabetes mellitus
Tadalafil	**Yes**	2003	Cmax = 378 µg/L Tmax = 2 h T1/2 = 17.5 h Not affected by heavy/fatty meals	ED: 10-20 mg on demand ED: 5 mg OD LUTS: 5 mg OD PAH: 40 mg	ED PAH LUTS	Headache: 14.5% Flushing: 4.1% Dyspepsia: 12.3% Nasal congestion: 4.3% Dizziness: 2.3% Back pain: 6.5% Myalgia: 5.7%		
Vardenafil	**Yes**	2003	Cmax = 18.7 µg/L Tmax = 0.9 h T1/2 = 3.9 h Affected by heavy/fatty meals	ED: 5–20 mg on demand	ED	Headache: 16% Flushing: 12% Dyspepsia: 4% Nasal congestion: 10% Dizziness: 2% Abnormal vision: < 2%		
Avanafil	**Yes**	2013	Cmax = 5.2 µg/L Tmax = 0.5–0.75 h T1/2 = 6–17 h Affected by heavy/fatty meals	ED: 50–200 mg on demand	ED	Headache: 9.3% Flushing: 3.7% Dyspepsia: uncommon Nasal congestion 1.9% Dizziness: 0.6% Back pain: < 2% Myalgia: < 2%		
Udenafil	**No**	2005	Cmax = 1137 µg/L Tmax = 0.76 h T1/2 = 9.88 h	ED: 100 mg on demand	ED	Headache: 2–9% Flushing: 11–23% Dyspepsia: uncommon Nasal congestion: 4–7% Red eye: 4–7% Chest discomfort: 0–5%		
Lodenafil	**No**	2007	Cmax = 157 µg/L Tmax = 1.2 h T1/2 = 2.4 h	ED: 80 mg on demand	ED	Headache: 15–22% Flushing: 5–6% Dyspepsia: 5–22% Nasal congestion: 5–11% Abnormal vision: 5–6%		
Mirodenafil	**No**	2011	Cmax = 2989 µg/L Tmax = 1.4 h T1/2 = 2.5 h	ED: 80 mg on demand	ED	Headache: 8–11% Flushing: 10–16% Dyspepsia: 3% Red eye: 3–4% Chest discomfort: 0–3%		
Benzamidenafil	**No**	-	ID	ID	ID	ID	ID	ID
Dasantafil	**No**	-	ID	ID	ID	ID	ID	ID
Icariin	**No**	-	ID	ID	ID	ID	ID	ID
Zaprinast	**No**	-	ID	ID	ID	ID	ID	ID

Abbreviations: AKI, acute kidney injury; BP, blood pressure; Cmax, serum maximum concentration; CHF, chronic heart failure; CKD, chronic kidney disease; CVD, cardiovascular disease; ED, erectile dysfunction; ID, insufficient data; NO, nitric oxide; NYHA, New York Heart Association; OD, once daily; PAH, pulmonary arterial hypertension; PDE5I, phosphodiesterase 5 inhibitor; Tmax, transport maximum.

## Abbreviations

ADQI: Acute Dialysis Quality Initiative; AKI, acute kidney injury; AKIN, Acute Kidney Injury Network; Anti-dsDNA, antibody to double stranded DNA; Bax, proapoptotic protein; Bcl-2, antiapoptotic gene; BP, blood pressure; bFGF, basic fibroblast growth factor; Cmax, serum maximum concentration; BMP-7, bone morphogenetic protein-7; cAMP, cyclic adenosine monophosphate; cGMP, cyclic guanosine monophosphate; CHF, chronic heart failure; CIN, contrast-induced nephropathy; CKD, chronic kidney disease; CM, contrast media; COX, cyclo-oxygenase; CVD, cardiovascular disease; CYP3A4, cytochrome P450 3A4; DNA, deoxyribonucleic acid; DOCA, deoxycorticosterone acetate; ED, erectile dysfunction; EMA, European Medicines Agency; eNOS, endothelial nitric oxide synthase; ERK, extracellular signal-regulated kinases; FDA, Food and Drug Administration; GTP, guanosine-5′-triphosphate; GC, guanylyl cyclase; GFR, glomerular filtration rate; GSH, glutathione; HEK, human embryonic kidney cells; HGF, hepatocyte growth factor; HIF-2a, hypoxia induced factor 2a; HO-1, heme oxygenase 1; HSP-70, Heat shock protein 70, huMSCs, human umbilical cord mesenchymal stem cells; ICAM-1, intercellular adhesion molecule-1; ID, insufficient data; IgG, immunoglobulin G; IL, interleukin; iNOS, inducible nitric oxide synthase; IR, ischemia reperfusion; KDIGO, Kidney Disease, Improving Global Outcomes; KIM-1, kidney injury molecule-1; KO, knockout; LNAME, N(ω)-nitro-L-arginine methyl ester; MeSH, medical subject headings; Mac387, macrophage antibody; MDA, malondialdehyde; NAC, N-acetylcysteine; NF-Kb, nuclear factor kappa light-chain-enhancer of activated B cells; NGAL, neutrophil gelatinase-associated lipocalin; NO, nitric oxide; NOS, nitric oxide synthase; Nrf-2, nuclear factor erythroid 2-related factor-2; NQO1, NADPH quinine oxidoreductase 1; NYHA, New York Heart Association; OD, once daily; PAH, pulmonary arterial hypertension; PDE5, phosphodiesterase 5; PDE5Is, phosphodiesterase 5 inhibitors; PGC-1a, peroxisome proliferator-activated receptor γ coactivator 1a; PKG, protein kinase G; PRISMA, preferred reporting items for systematic reviews and meta-analyses; RIFLE, risk, injury, failure, loss and end-stage kidney disease; ROS, reactive oxygen species; SBP, systolic blood pressure; sCr, serum creatinine; Tfam, mitochondrial transcription factor; TGF-β1, transforming growth factor beta; Tmax, transport maximum; TSP, thrombospondin; TUNEL, terminal deoxynucleotidyl transferase dUTP nick end labeling; TNF-a, tumor necrosis factor -a; VEGF, vascular endothelial growth factor; WT-1, Wilms’ tumor 1 gene.

## Appendix A. Literature Search Strategy

Database: Ovid Medline Epub Ahead of Print, In-Process & Other Non-Indexed Citations, Ovid MEDLINE(R) Daily and Ovid MEDLINE(R) 1946 to November 2019.

Search Strategy:

((((((((((((PDE5i[Title/Abstract]) OR avanafil[Title/Abstract]) OR benzamidenafil[Title/Abstract]) OR dasantafil[Title/Abstract]) OR icariin[Title/Abstract]) OR lodenafil[Title/Abstract]) OR mirodenafil[Title/Abstract]) OR sildenafil[Title/Abstract]) OR tadalafil[Title/Abstract]) OR vardenafil[Title/Abstract]) OR udenafil[Title/Abstract]) OR zaprinast[Title/Abstract]) AND (((((((((((((((((((((((((renal[Title/Abstract]) OR kidney[Title/Abstract]) OR nephrotoxicity[Title/Abstract]) OR contrast[Title/Abstract]) OR CIN[Title/Abstract]) OR AKI[Title/Abstract]) OR nephrotoxic[Title/Abstract]) OR cisplatin[Title/Abstract]) OR aminoglycoside[Title/Abstract]) OR trauma[Title/Abstract]) OR acute kidney injury[Title/Abstract]) OR nsaids[Title/Abstract]) OR non steroidal[Title/Abstract]) OR shock[Title/Abstract]) OR sepsis[Title/Abstract]) OR hypoperfusion[Title/Abstract]) OR hypovolaemia[Title/Abstract]) OR renal artery stenosis[Title/Abstract]) OR obstruction[Title/Abstract]) OR acute tubular necrosis[Title/Abstract]) OR glomerulonephritis[Title/Abstract]) OR nephritis[Title/Abstract]) OR renal failure[Title/Abstract]) OR cyclosporine[Title/Abstract]) OR adenine[Title/Abstract]).

## Appendix B. Animal Studies in the AKI-CKD Transition Spectrum (Focusing on Renal Function and/or Structure Alterations for up to Three Months, Not Fulfilling the KDIGO Definition for CKD [6])

**Table A1:** Animal studies evaluating the potential reno-protective effects of sildenafil.

Reference /Country/Year	Studied Animal	Model	PDE5I Route	Timing	Sample	Renal Injury Effects	PDE5I Renal Effects	Outcome
[81]/ Venezuela/2005	Male Sprague Dawley rats	5/6 nephrectomy	Sildenafil Orally 2.5 mg/kg/day Either immediately after nephrectomy for 8 weeks Or 4 weeks after nephrectomy for 4 weeks	POST	8 weeks	↑sCr, ↑SBP, ↑proteinuria ↓urinary NOX, ↓cGMP Glomerulosclerosis Tubulo-interstitial damage Macrophage accumulation Increased number of apoptotic cells	↓sCr, ↓SBP, ↓proteinuria ↑ urinary NOX, ↑cGMP All changes improved especially if PDE5i was given early	POS
[82]/ Spain/2007	Laboratory large-white pigs	Right single nephrectomy after 45 min of vascular clamping + Auto-transplantation + Left nephrectomy	Sildenafil Orally 100 mg, 2 h pre-op	PRE	0/15/30/45/60 min after unclamping	↓RVF, ↑RVR, ↓NO Minimal differences in tubular and endothelial structure	↑↓RVF, ↓RVR, ↑NO Minimal differences in tubular and endothelial structure	POS
[83]/ Egypt/2008	Adult male Wistar albino rats	L-NAME Orally 50 mg/kg/day for 4 weeks	Sildenafil Orally 5 mg/kg/day 2 weeks after L-NAME for 2 weeks	POST	At 4 weeks	↓NO, ↓cGMP Glomerular collapse/mesangial matrix expansion with minimal cellular proliferation	↑NO, ↑cGMP Improvement in histological alterations	POS
[84]/ Spain/2009	Laboratory minipigs	Right single nephrectomy after 45 min of vascular clamping +Auto-transplantation +Left nephrectomy	Sildenafil Orally 100 mg, 1.5 h pre-op	PRE	0/15/30/45/60 min after unclamping	↓RVF, ↓RVR	↑RVF, ↓ RVR, ↑NO Reduced tubular edema, Improved endothelial cell integrity and mitochondrial ultrastructure	POS
[85]/ Korea/2009	Male Sprague Dawley	Streptozotocin Single intravenous dose 60 mg/kg	Sildenafil Orally 3 mg/kg/day For 8 weeks	POST	At 8 weeks	↑glucose, ↑urine output ↑urine 8-OH dG ↑urine albumin ↑Kidney/BW ratio, ↑iNOS ↑Nitrotyrosine, ↑MCP-1 ↑ED-1	↑glucose, ↓urine output ↓urine 8-OH dG ↓urine albumin ↓Kidney/BW ratio, ↓iNOS ↓Nitrotyrosine, ↓MCP-1, ↓ED-1	POS
[86]/ Korea/2012	Male Sprague Dawley rats	Unilateral Nephrectomy and 1 week later DOCA strip 200 mg/kg implantation	Sildenafil Orally 50 mg/kg/day 2 weeks after DOCA for 2 weeks	POST	At 4 weeks	↑mortality, ↑SBP, ↑ Kidney weight ↓CrCl, ↑sCr, ↑FeNa, ↑ACR ↑ED-1, ↑BAX, ↓Bcl2, ↑aSMA, ↑TGF-b1 ↑TUNEL +ve, ↑fibronectin ↑mRNA TGF-β1/MCP-1 ↑ICAM 1t Tubular casts/Tubular obstruction/Vessel dilatation/Glomerulosclerosis/interstitial expansion	↓mortality, ⇔ SBP, ↓Kindey weight ↑CrCl, ↓sCr, ↓FeNa, ↓ACR ↓aSMA ↓ED-1, ↓BAX, ↑Bcl2, ↓TGF-b1 ↓TUNEL +ve, ↓fibronectin ↓mRNA TGF-β1/MCP-1/↓ mRNA ICAM1 Reversed all renal injuries	POS
[87]/ Venezuela/2012	Male Wistar rats	5/6 nephrectomy	Sildenafil Orally 5 mg/kg/day for 60 days 24 h after nephrectomy	POST	Every 2 weeks	↑Kidney hypertrophy ↑Proteinuria, ↓NO2/NO3 ↓GMP (urine) ↑Nitrotyrosine	↓Kidney hypertrophy ↓Proteinuria, ↑NO2/NO3 ↑cGMP (urine) ↓Nitrotyrosine	POS
[19]/ Egypt/2013	Male Wistar rats	Cyclosporine A Subcutaneously 20 mg/kg/day 21 days	Sildenafil Orally 5 mg/kg/day 21 days	POST	At 21st day–urine sample/blood sample/renal tissue excision	↑BUN, ↑sCr, ↑MDA levels ↑Urine Albumin/Cr ↓GSH/NO/catalase activity ↑iNOS, TNF-a, Caspase 3 activity Tubular degeneration and dilation and necrosis/Glomerulat damage/Congestion Dilated Bowman’s space/Hemorrhage	↓BUN, ↓sCr, ↓MDA levels ↓Urine Albumin/Cr, ↑eNOS ↑GSH/NO/catalase activity ↓iNOS, TNF-a, Caspase 3 activity Improved all histological changes	POS
[88]/ United Kingdom/2014	Porcine kidneys	20 min warm ischemia followed by 2 or 18 h of cold storage	Sildenafil Intravenously 1.4 mg/kg 10 min prior to injury and 20min after reperfusion	PRE and POST	Samples during reperfusion	↓RBF, ↑intrarenal resistance ↓Urine cGMP ↑ sCr Steady increase of K+ ↑Tubular injury No difference in groups Tubular dilatation and debris and interstitial edema/Ischemic changes	↑RBF, ↓intrarenal resistance ↑Urine cGMP, ↓sCr No significant difference in K+ No effect on tubular injury regarding GAL/Endothelin1 Slight improvement of histological	POS
[89]/ Brazil/2014 [90]/ Brazil/2014	C57BL/6 mice	Left renal artery clamping for 2 weeks	Sildenafil Orally 40 mg/kg/day 2 weeks post op for 2 weeks	POST	4 weeks	Left kidney atrophy (clipped) Right kidney hypertrophy ↓BW, ↑SBP, ↑HR ↑Intrarenal angiotensin I/II ⇔Plasma angiotensin I/II/1-7 ↓NO, ↑ONOO-, ↑O2- Impaired vasodilation response to Ach	↓Left kidney atrophy ↓Right kidney hypertrophy Normal BW, ↓SBP, ↓HR ↓Intrarenal angiotensin I/II ↑Plasma angiotensin 1-7 ↑NO, ↓ONOO-, ↓O2- Normal vasodilation response to Ach	POS
[91]/ Egypt/2016	White albino male rats	Streptozocin Single intraperitoneal dose 55 mg/kg	Sildenafil Orally 3 mg/kg/day For 8 weeks after Diabetic nephropathy	POST	8 weeks	↓SOD, ↑TGF-β1, ↓NO, ↑sCr ↑BUN, ↑proteinuria ↑Kidney IL-1β ↑Advanced glycation end products (AGEPs)	↑SOD, ↓TGF-β1, ↑NO, ↓sCr ↓BUN, ↓proteinuria ↓Kidney IL-1β ↓Advanced glycation end products (AGEPs)	POS
[92]/ India/2016	Sprague-Dawley rats	Streptozocin Single intraperitoneal dose 60 mg/kg	Sildenafil Orally 2.5 mg/kg/day for 6 weeks after 28 days	POST	At 28th and 70th day	↑sCr, ↑BUN, ↓CrCl ↑Total protein excretion ↑albumin (urine) Bowman’s capsule thickening, glomerular sclerosis	↓sCr, ↓BUN, ↑CrCl ↓Total protein excretion ↓albumin (urine) Histopathology improvement	POS
[93]/ Italy/2017	Male CD-1 mice	Streptozotocin Single intraperitoneal dose 150 mg/kg	Sildenafil Intraperitoneally 1.6 mg/kg 3 days after STZ, for 4 weeks	POST		↑Glucose (urine), ↑MAP, ↓GFR ↑urinary ACR, ↑NGAL, ↑RRI ↓Renal volume, ↓BMP7 ↑suPAR, ↑Vascular leakage ↑FITC-dextran extravasation Reduced glomerular diameter/focal and segmental hyperplasia with diffuse mesangial proliferation/increased mesangial matrix deposition/acute tubular degeneration/eosinophilia/proximal tubule basal membrane thickening	↓Urine glucose, ↓MAP, ↑GFR ↓urinary ACR, ↓NGAL, ↓RRI ↑Renal volume, ↑BMP7, ↓miR-22 ↓suPAR, ↓Vascular leakage ↓FITC-dextran extravasation Reduced mesangial matrix deposition	POS
[94]/ Egypt/2017	Adult male Sprague-Dawley rats	Doxorubicin Intraperitoneally 3.5 mg/kg Twice weekly for 3 weeks	Sildenafil Orally 5 mg/kg/day for 21 days	POST		↑Urea, ↑sCr, ↑uric acid ↑MDA, ↓GSH, ↑TNF-a ↑caspase-3 Eosinophilic casts, tubule degeneration, vacuolization, endothelial cell edema	↓Urea, ↓sCr, ↓uric acid ↓MDA, ↑GSH, ↓TNF-a ↓caspase-3 Histological improvement	POS
[95]/ South Africa/2017	Nulliparous pregnant female Sprague-Dawley rats	L-NAME Orally 0.3 g/L (drinking water) 4-8 days for EOPE + 8-14 days for LOPE	Sildenafil Orally 10 mg/kg 4-8 days for EOPE 8-14 days for LOPE	POST	Gestational Day 19	↑BP, ↑Urine excretion ↑Urinary nephrin mRNA ↑Podocin (urine), ↑sFlt-1(mRNA) ↓VEGF (mRNA), ↓PIGF Glomerular and tubular damage and mononuclear cell infiltration	↓BP ↓Urinary nephrin mRNA ↓Podocin (urine) ↓sFlt-1 (mRNA) ↑VEGF (mRNA), ↑PIGF levels Attenuated histopathological changes	POS
[96]/ Netherlands/2017	Rats	Adriamycin Or Streptozocin	Sildenafil Orally 5 mg/kg/day for 6 weeks	POST	Immortalized mouse podocytes + Mouse kidney cortex	↑TRPC6 expression ↓Nephrin, ↑Glomerular desmin ↑Urinary albumin ↑Glomerular lesions	↓TRPC6 expression, ↓Ca2+ influx ↑Nephrin ↓Glomerular desmin ↓Urinary albumin	POS
[97]/ Oman/2018	Male Sprague-Dawley rats	Adenine (0.25% w/w) orally Daily for 5 weeks	Sildenafil Orally (0.1, 0.5 or 2.5 mg/kg) Daily for 5 weeks (alone or concomitantly with adenine)	SIM	At Day 5	↑BUN, sCr, uric acid, P, NGAL, ↑total NO, IS, Caspase 3 +ve cells ↑Albumin, NAG activity ↓Osmolality, CrCl in urine ↓CAT, glutathione reductase, SOD ↓TAC, ↑MAPK ↑Fibrosis ↑Adiponectin, cystatin-C, TNF-a ↓Sclerostin, ↑MDA Tubular necrosis, tubular dilatation, tubular cast formation, necrotic nuclei, tubular cells apoptosis, cellular shedding, mononuclear infiltration	↓BUN, sCr, uric acid, ↓P, NGAL, ↓total NO, IS, ↓Caspase 3 +ve cells ↓Albumin, ↓ NAG activity ↑Osmolality, ↓CrCl in urine ↑CAT, SOD ↓glutathione reductase, ↑TAC, ↓MAPK, ↓Fibrosis ↓Adiponectin, cystatin-C, TNF-a ↑Sclerostin (not 0.1 mg/kg) ↓MDA Improved tubular necrosis, tubular dilatation, tubular cast formation, mononuclear infiltration	POS
[98]/ Egypt/2018	Male albino rats Sprague-Dawley	Streptozotocin Single intraperitoneal dose 45 mg/kg	Sildenafil Orally: 3 mg/kg/Day 3 weeks after STZ for 15 days	POST	Day 16 after initiation of Sildenafil	↑sCr, ↑BUN ↑fasting and post prandial glucose ↓insulin levels ↑insulin resistance ↑MDA, ↓GSH, ↓CAT, ↓GPx, ↓SOD, ↓TAC	↓sCr, ↓BUN ↓fasting and post prandial glucose ↑insulin levels ↓insulin resistance (insignificant) ↓MDA, ↑GSH, ↑CAT, ↑GPx, ↑SOD, ↑TAC	POS

Abbreviations: AKI, acute kidney injury; ACR, albumin-creatinine ratio; aSMA, α-smooth muscle actin; Bax, proapoptotic protein; Bcl-2, antiapoptotic gene; BP, blood pressure; BUN, blood urea nitrogen; BW, body weight; Ca^2+^, calcium; CAT, catalase; cGMP, cyclic guanosine monophosphate; CrCl, creatinine clearance, ED-1, monoclonal antibody, eNOS, endothelial NOS, FeNa, fractional excretion of sodium, FITC, fluorescein isothiocyanate, GFR, glomerular filtration rate; GPx, glutathione peroxidase; GSH, glutathione; HR, heart rate; ICAM-1, intercellular adhesion molecule 1; IL, interleukin; IS, indoxyl sulfate; iNOS, inducible NOS; K, potassium; MAP, mean arterial pressure; MAPK, mitogen-activated protein kinase; MCP-1, monocyte chemoattractant protein 1; MDA, malondialdehyde; Na, sodium; NAG, N-acetyl-beta-D-glucosaminidase; NGAL, neutrophil gelatinase-associated lipocalin; NO, nitric oxide; NOX, NADPH oxidase; P, phosphorus; PDE5I, phosphodiesterase 5 inhibitor; PIGF, placenta growth factor; RRI, renal resistive index; RVF, renal vascular flow; RVR, renal vascular resistance; sCr, serum creatinine; sFlt1, soluble fms-like tyrosine kinase-1; SOD, superoxide dismutase; SBP, systolic blood pressure; suPAR, soluble urokinase-type plasminogen activator receptor; TAC, total antioxidant capacity; TGF-β1, transforming growth factor beta 1; TRPC6, transient receptor potential cation channel 6; TNF-a, tumor necrosis factor a; TUNEL, terminal deoxynucleotidyl transferase dUTP nick end labeling; VEGF, vascular endothelia growth factor; 8-OH dG, 8-hydroxydeoxyguanosine; ↓, reduced; ↑, increased.

**Table A2:** Animal studies evaluating the potential reno-protective effects of tadalafil.

Reference/ Country/Year	Studied Animal	Model	PDE5I Route	Timing	Sample	Renal Injury Effects	PDE5I Renal Effects	Outcome
[99]/ Turkey/2013	Male Sprague Dawley rats	SWL model	Tadalafil *Orally: *1 mg/kg Single dose 150 min prior to SWL	PRE	Nephrectomy at Day 1/3/7	Loss of micro-villi Tubular degeneration and necrosis Interstitial edema and fibrosis ↑ HSP-70	Reduced all histological damage ↓HSP-70	POS
[100]/ Turkey/2017	Male Sprague Dawley	SWL model	Tadalafil *Orally: *1 mg/kg 60 min prior to SWL	PRE	Bilateral nephrectomy 7 days post SWL	Renal tubular damage Peritubular fibrosis/Loss of microvilli ↑HSP-70	Significantly less tissue damage ⇔HSP-70 (glomerular) ↓HSP-70 (medullar/cortical)	POS

Abbreviation: AKI, acute kidney injury; HSP-70, heat shock protein 70; PDE5I, phosphodiesterase 5 inhibitor; SWL, shock wave lithotripsy; ↓, reduced; ↑, increased ⇔, no change.

**Table A3:** Animal studies evaluating the potential reno-protective effects of icariin.

Reference/ Country/Year	Studied Animal	Model	PDE5I Route	Timing	Sample	Renal Injury Effects	PDE5I Renal Effects	Outcome
[101]/ China/2011	Male Sprague–Dawley rats	Streptozotocin Single Dose Intravenously 40 mg/kg	Icariin Orally 80 mg/kg For 8 weeks From 5th to 20th week post streptozotocin	POST	Day 7 and Week 13	↑sCr, ↑BUN, ↑Glucose, ↑MDA, ↑Hyp, ↓SOD, ↑Collagen IV, ↑TGF-β1 Glomerular Hypertrophy Expansion of mesangial area and ECM	↓sCr, ↓BUN, ↓MDA, ↓Hyp ↑SOD, ↓Collagen IV, ↓TGF-β1 Inhibited these changes	POS
[102]/ Chiana/2014	Male Sprague-Dawley rats	5/6 right nephrectomy model	Icariin *Orally* 20 + 40 mg/kg/day 1 week after AKI for 12 weeks	POST	24 h before AKI and at Week 12	↑BUN,↑sCr, ↑ urinary protein ↑Apoptotic rate, ↑Bcl-2, ↑Bax ↓G0/G1 phase cells ↑S phase cells	↓BUN, ↓sCr, ↓urinary protein ↓Apoptotic rate, ↓Bcl-2, ↓Bax ⇔G0/G1 phase cells, ↑G2/M phase ↓ S phase cells	POS
[103]/ China/2015	Male Sprague Dawley rats	1st stage: Partial nephrectomy 2nd stage: Right renal ligation	Icariin *Orally* 40 mg/kg/day 8 weeks	POST	At 8 weeks	↑BUN, ↑sCr, ↑uric acid Mesangial expansion/Edema Basement membrane thickening and capillary compression/occlusion. Glomerular sclerosis/fibrosis Inflammatory cell infiltration	↓BUN, ↓sCr, ↓uric acid ↑Renal progenitor cell proliferation ↓TGF-β1 Significantly improved glomerular lesions and blunted rest of the changes	POS
[22]/ China/2017	Female Wistar rats	Pregnancy induced hypertension L-NAME 0.5 g/L from Day 12 of gestation	Icariin *Orally* 10/50/100 mg/kg 18 days of gestation	POST	BP: Days 1 and 18 Kidney tissue: Day 18	↑SBP (Day 18), ↑ BUN, ↑sCr ↑Proteinuria, ↓Pup weight ↓Nephrinm, ↑Ang II, ↑AGT Mesangial expansion Basement membrane thickening	↓SBP (high dose), ↓BUN, ↓sCr ↓Proteinuria (medium/high dose) No difference in pup weight ↑Nephrin, ↓Ang II, ↓AGT Markdely reduced severity of lesions	POS
[104]/ China/2018	MRL/lpr mice	K/O mice	Icariin Orally 10 mg/kg/day 8 weeks	POST	Every 2 weeks	↑Urine protein,↑IgG deposit ↑sCr, ↑BUN, ↑TNF-a ↑Serum anti-dsDNA ↑Translocation and phosphorylation of NF-kBp65 ↑F4/80, ↑NLRP3, ↑caspase 1p20 Increased glomerular proliferation/sclerosis/peripheral inflammation	↓Urine protein, ↓IgG deposit ↓sCr, ↓BUN, ↓TNF-a ↓Serum anti-dsDNA ↓Translocation and phosphorylation of NF-kBp65 ↓F4/80, ↓NLRP3, ↓caspase 1p20 Improved all changes	POS

Abbreviations: AKI, acute kidney injury; Ang II, angiotensin II; Anti-dsDNA, antibody to double stranded DNA; AGT, angiotensinogen; Bax, proapoptotic protein; Bcl-2, antiapoptotic gene; BP, blood pressure; BUN, blood urea nitrogen; F4/80, macrophage marker; Hyp, hydroxyproline; IgG, immunoglobulin G; MDA, malondialdehyde; PDE5I, phosphodiesterase 5 inhibitor; SBP, systolic blood pressure; sCr, serum creatinine; SOD, superoxide dismutase; TGF-β1, transforming growth factor beta 1; TNF-a, tumor necrosis factor a; ↓, reduced; ↑, increased.

**Table A4:** Animal studies evaluating the potential reno-protective effects of vardenafil.

Reference/ Country/Year	Studied Animal	Model	PDE5I Route	Timing	Sample	Renal Injury Effects	PDE5I Renal Effects	Outcome
[105]/ Germany/2008	Sprague Dawley rats	Mouse monoclonal anti-Thy 1 antibody ER-4 Single injection, 1 mg/kg	Vardenafil *Orally* 20 mg/kg within 18 h and 10 mg/kg/day for 48 h	PRE and POST	24-h urine collection on Days 2 and 6 Blood sample: Day 6	↑PDE5-A, ⇔sCr ↑proteinuria Mesangial proliferation	↑cGMP, ↓TSP-1, ⇔sCr ↓proliferation/cell number(glomerular) ↓collagen IV/fibronectin (glomerular) ↓TGF-β activation ⇔proteinuria	POS
[106]/ China/2009	New Zealand Rabbits	Invagination of ureter in renal pelvis	Vardenafil *Orally* 0.3 mg/kg/day For 8 weeks post op	POST	8 weeks	Dilated renal pelvises Fibrotic PUJ ↑TGF-β1 ↓nNOS	Dilated renal pelvises Less fibrotic PUJ ↓TGF-β1 ↑nNOS	POS
[107]/ Hungary/2013	Sprague Dawley male rats	Streptozotocin Single intraperitoneal dose 60/mg/kg	Vardenafil *Orally* 10 mg/kg/day for 8 weeks 72 h post STZ	POST	8 weeks after AKI	↓cGMP, NCS elevated Urea levels Decreased body weight No difference in MAP ↑Urine protein/creatinine ratio ↑Fibronectin, ↑TGF-β1, ↑desmin, ↓nephrin, ↑Nitrotyrosine, ↑NOS Glomerular hypertrophy Mesangial expansion Adhesions to Bowman’s capsule Tubular dilatation and atrophy Mononuclear cell infiltration	↑cGMP Developed kidney hypertrophy No difference in MAP ↓Urine protein/creatinine ratio ↓Fibronectin, ↓TGF-β1 ↓desmin, ↑nephrin No difference Attenuated all changes	POS
[108]/ Turkey/2015	Male Swiss albino mice	Cyclosporine A 30 mg/kg Subcutaneously Daily for 28 days	Vardenafil *Orally* 30 mg/kg/day For 28 days	PRE	At 28 days	↓Kidney weight ↑BUN, ↑sCr, ↑TOS levels ↓TAS levels,↓tissue NO ↓COX-1, ↓COX-2, ↓TGF-β1 ↓Pgp levels, ↓PDGF-A, ↓PDGF-C Histological changes: cortex/outer medulla	No change in kidney weight ↓BUN, ↓sCr, ↓TOS levels ↑TAS levels, ↑tissue NO ↑COX-1, ↑COX-2, ⇔TGF-β1 ↑Pgp levels, ↑PDGF-A, ↑PDGF-C Normal histopathological appearances	POS

Abbreviations: AKI, acute kidney injury; BUN, blood urea nitrogen; cGMP, cyclic guanosine monophosphate; COX, cyclo-oxygenase; FeNa, fractional excretion of sodium; MAP, mean arterial pressure; NCS, not clinically significant; NO, nitric oxide, nNOS, neuronal NOS; PDE5I, phosphodiesterase 5 inhibitor; PDGF, platelet-derived growth factor; Pgp, P glycoprotein; PUJ, pelvic ureteric junction; sCr, serum creatinine; TAS, total antioxidant status; TGF-β1, transforming growth factor beta 1; TOS, total oxidant status; TSP-1, thrombospondin -1; ↓, reduced; ↑, increased ⇔, no change.

**Table A5:** Animal studies evaluating the potential reno-protective effects of zaprinast and udenafil.

Reference/ Country/Year	Studied Animal	Model	PDE5I Route	Timing	Sample	Renal Injury Effects	PDE5I Renal Effects	Outcome
[109]/ Japan/1998	Mongrel dogs	Cut left renal nerves and electrostimulation of left renal bundle (distal end)	Zaprinast *Intra-renal arterial infusion* 10 or 100 μg/kg/min	SIM	Simultaneously	↓Urine flow, ↓U_Na_V, ↓FeNa ⇔RBF, ⇔GFR	↑Urine flow, ↑U_Na_V, ↑FeNa ⇔ RBF, ⇔GFR, ↓RVR ↑Renal venous cGMP	POS
[110]/ Korea/2010	10-week-old male Sprague-Dawley	Right nephrectomy + Left renal artery clamping for 45 min and Cyclosporine A 15 mg/kg subcutaneously	Udenafil *Orally: 10 mg/kg* For 28 days after the procedure	SIM and POST	On Day 28 blood samples and left nephrectomy	↑BUN, ↑sCr, ↓eNOS, ⇔VEGF Decreased thickness of the proximal tubules and nuclei, vacuolization of the cytoplasm, altered cellular shape, fewer nuclei	↓BUN, ↓sCr, ↑eNOS, ⇔VEGF ↓VEGF mRNA	POS

Abbreviations: AKI, acute kidney injury; BUN, blood urea nitrogen; cGMP, cyclic guanosine monophosphate; eNOS, endothelial NOS; FeNa, fractional excretion of sodium; GFR, glomerular filtration rate; PDE5I, phosphodiesterase 5 inhibitor; RBF, renal blood flow; RVR, renal vascular resistance; sCr, serum creatinine; UNaV, urinary sodium excretion; VEGF, vascular endothelia growth factor; ↓, reduced; ↑, increased ⇔, no change.
